# Designing High-Performance Dual-Ion Batteries at High-Voltage: Challenges, Strategies, and Prospects

**DOI:** 10.1007/s40820-026-02114-x

**Published:** 2026-04-21

**Authors:** Chong Han, Yan-Song Xu, Ziyang Hu, An-Min Cao, GuanHua Chen

**Affiliations:** 1 Department of Chemistry, The University of Hong Kong, Pok Fu Lam Road, Hong Kong, 999077 People’s Republic of China; 2 College of Chemistry, Huazhong Agricultural University, Wuhan, 430070 People’s Republic of China; 3 Hong Kong Quantum AI Lab Limited, Hong Kong, People’s Republic of China; 4 CAS Key Laboratory of Molecular Nanostructure and Nanotechnology, and Beijing National Laboratory for Molecular Sciences, Institute of Chemistry, Chinese Academy of Sciences (CAS), Beijing, 100190 People’s Republic of China

**Keywords:** Dual-ion batteries, Cathode materials, Anion intercalation chemistry, Electrolyte design, Electrode–electrolyte interphase

## Abstract

Dual-ion batteries (DIBs) have emerged as a promising next-generation energy storage technology with compelling advantages induced by anion intercalation mechanism, including high operating voltage (> 4.8 V), abundant raw materials of dual-carbon electrodes, intrinsic electrode safety, and environmental friendliness. However, this mechanism at the cathode side leads to new challenges different from conventional lithium-ion batteries, such as high-voltage electrolyte decomposition, solvent co-intercalation, and limited capacity. In this review, beyond the fundamental understanding and recent progress of DIBs, we systematically discuss their challenges, such as electrolyte decomposition at high voltages, solvent co-intercalation, interfacial instability, asymmetric electrode kinetics, and limited capacity. Corresponding strategies are then summarized, ranging from the design of electrolytes with high oxidation resistance and anion solvation microenvironment regulation to electrode–electrolyte interfacial construction and the discovery of cathode materials with high capacity. The strategies for dynamic matching between cathode and anode, as well as designing more reliable dual-carbon full cells, are further discussed. Finally, prospects for future research are outlined, with emphasis on advanced characterization technologies, theoretical modeling, and rational design of electrode and electrolyte systems. This review provides a roadmap for advancing high-voltage DIBs toward practical applications in large-scale and sustainable energy storage.

## Highlights

- Clarifies fundamental anion intercalation mechanisms, voltage window and solvation microenvironment in high-voltage dual-ion batteries.
- Systematically identifies key challenges: electrolyte decomposition, solvent co-intercalation, unstable interphases, kinetic mismatch, limited capacity, low-temperature and safety issues.
- Summarizes electrolyte, electrode, and interfacial engineering strategies and outlines future directions in advanced characterization, theory and artificial intelligence-guided materials/electrolyte design for practical dual-ion batteries deployment.

## Graphical abstract

**Figure:**
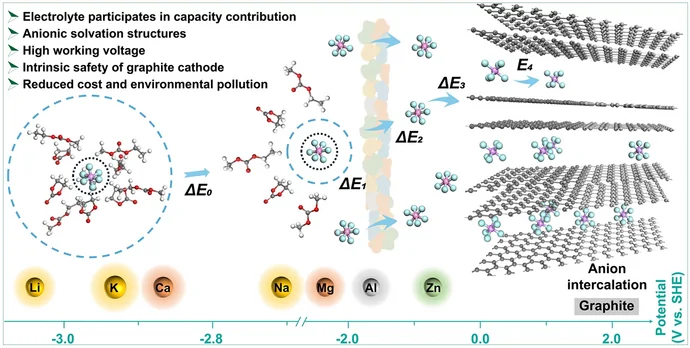

## Introduction

Growing risks to global energy shortages and the imperative to mitigate climate change have intensified the demand for renewable energy technologies, such as solar, wind, and tidal power. Nevertheless, the long-term reliable supply of these energy systems is suffering from their intermittent nature, thus developing advanced large-scale energy storage technologies is critical for effective grid integration and stable utilization. Secondary battery systems, represented by LIBs, have shown advantages such as high conversion efficiency, high energy density, and long cycle life, which promote its widespread commercialization in portable electronics, biomedical devices, electric vehicles, artificial intelligence (AI), and other fields [1, 2]. However, the sustainability of LIBs is being challenged by limited lithium reserves in Earth’s crust and its severely uneven distribution, coupled with the environmental risk or high price of key elements used in electrodes, such as nickel (Ni), and cobalt (Co) [3–5]. These challenges have accelerated researches toward next-generation secondary battery systems. Recently, those batteries such as sodium-ion batteries (SIBs), potassium-ion batteries (PIBs), and DIBs have gained increasing concern and are considered promising competitors for large-scale energy storage due to their advantages stem from abundant resource reserve and potential low-cost [6–9].

Unlike the conventional “rocking-chair” mechanism of LIBs/SIBs/PIBs, DIBs typically employ a distinct operating principle where both electrodes actively participate in ionic storage: graphite serves as the cathode while graphite or hard carbon functions as the anode [10, 11]. During charging, anions (e.g., TFSI^−^, FSI^−^, PF_6_^−^) from electrolyte intercalate into the cathode while cations (e.g., Li^+^, Na^+^, K^+^) are accommodated in the anode. Upon discharge, these ions, being extracted from the cathode and anode, return to the electrolyte, respectively. This unique mechanism brings the following advantages: (1) Enhanced energy density: the anion intercalation in the graphite cathode occurs at high operating voltages (e.g., ≥ 4.8 V vs. Li^+^/Li for Li^+^ as the working cation), substantially increasing the battery’s theoretical energy density; (2) Superior rate performance: the weak interactions between anions and solvent molecules, combined with a low diffusion energy barrier (~ 0.2 eV) of anions in graphite interlayers, enable rapid ionic diffusion kinetics and excellent rate capability. (3) Improved safety: the oxygen-free redox chemistry in the graphite cathode eliminates the risk of hazardous O_2_ release under high voltages, enhancing the inherent safety of the battery system. (4) Environmental and economic benefits: the use of metal-free graphite reduces both raw material costs and environmental impact, making DIBs particularly promising for practical applications in next-generation energy storage systems [12–14].

**Fig. 1:**
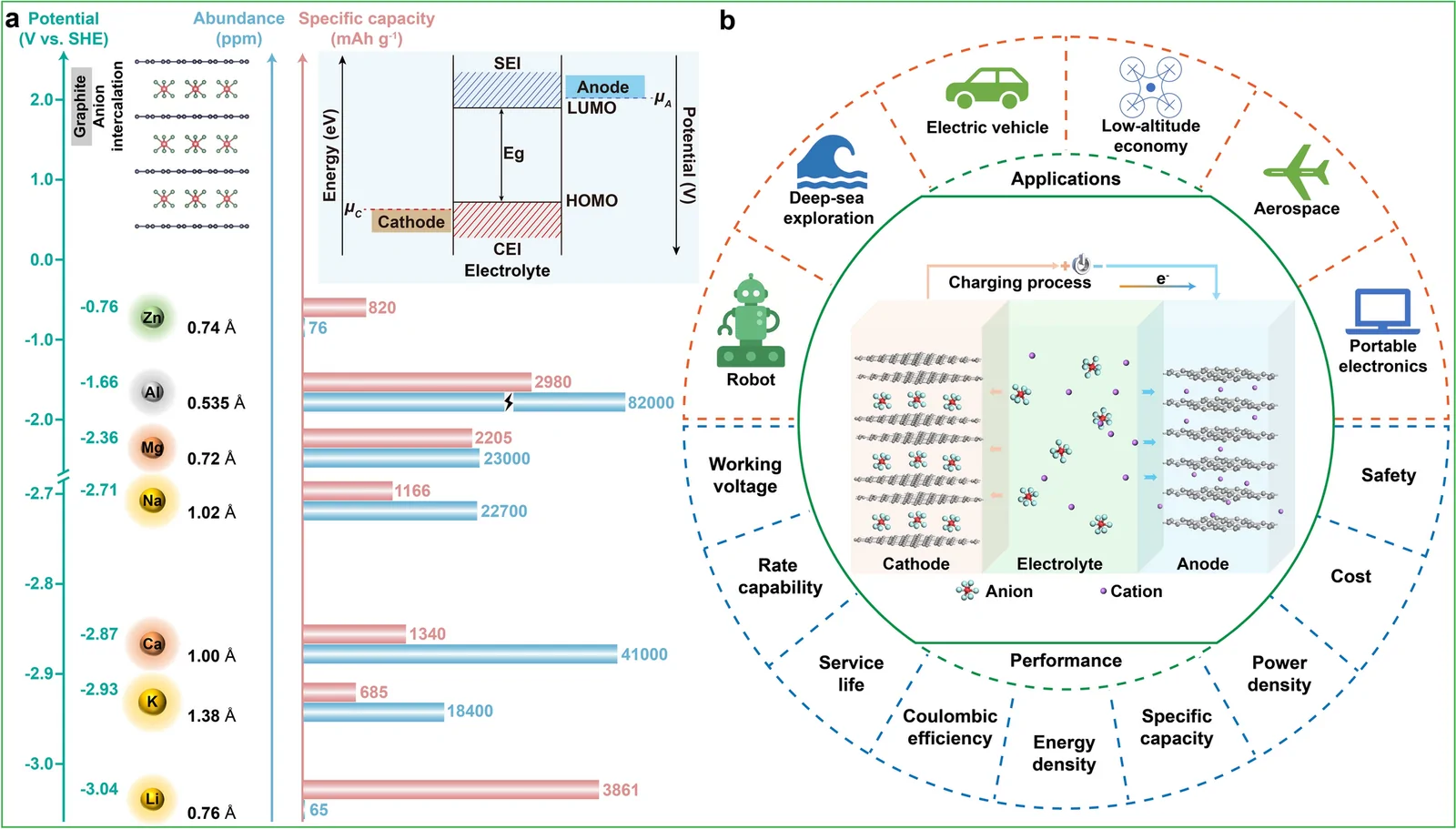
**a** A comparative comparison of various cations, containing some key parameters, such as Shannon ionic radius, redox potential, crustal abundance, and theoretical specific capacity.** b** Systematic summary of DIBs, including working mechanism, key electrochemical parameters, and potential future applications

The history of DIBs can be traced back to 1938, when Rüdorff and Hofmann observed the reversible intercalation of HSO_4_^−^ anions into graphite within an aqueous electrolyte based on concentrated sulfuric acid [15]. Despite the early uncovering of the anion intercalation mechanism, DIBs encountered a slow development period lasting for decades. Not until 2000, when Dahn and Seel’s groups systematically investigated the intercalation behavior of PF_6_^−^ into graphite in various electrolytes including ethyl methyl sulfone and carbonate mixtures, DIBs gradually gained the attention of researchers again [16, 17]. In 2012, Winter et al. proposed the concept of “dual ion battery”, which has been widely adopted and extensive efforts have been transferred to understand anion intercalation mechanisms, and explore suitable electrolytes and electrode materials for DIBs [18, 19]. The selection of cationic carriers (including Li^+^, Na^+^, K^+^, Mg^2+^, Ca^2+^, Zn^2+^, and Al^3+^) in electrolytes enables the construction of different metal-ion-based DIBs, which can combine the inherent advantages of DIBs and conventional metal-ion batteries. Typically, monovalent alkali metals such as Li, Na, and K deliver more negative redox potentials (− 3.04 V for Li/Li^+^, − 2.71 V for Na/Na^+^, and − 2.93 V for K/K^+^, all versus standard hydrogen electrode), which are critical for high energy density. [20] Meanwhile, as shown in Fig. 1a, multivalent cations (Mg^2+^, Ca^2+^, Zn^2+^, and Al^3+^) can store multiple electrons during their intercalation process, theoretically contributing higher specific capacity, but their less negative redox potential would impair it [21]. Thus, in this review, we will limit our discussion on alkali ions-based DIBs. Due to their potential to address major global challenges, DIBs were selected among the “Top Ten Emerging Technologies in Chemistry 2020” by the International Union of Pure and Applied Chemistry (IUPAC).

Very recently, Wang et al. demonstrated that difluoro 2,2-difluroethyl acetate (DFEA)-based electrolyte have a high ionic conductivity and remarkable electrochemical stability (up to 5.5 V vs. Li^+^/Li). DFEA can weaken the anion-solvent interactions, consequently reducing anion de-solvation kinetic barrier and suppressing solvent co-intercalation into graphite cathode [22]. Nonetheless, DIBs are still in their initial stage and face many challenges such as low Coulomb efficiency induced by irreversible electrolyte decomposition, solvent co-intercalation at high voltage, and relatively low theoretical capacity. Therefore, more fundamental research is needed that not only focuses on investigating the working mechanism and charge contribution mechanism but also on designing electrolytes with remarkable oxidation resistance and electrode materials with high theoretical capacity and robust structure, as well as on overcoming the challenges induced by assembling full cells (Fig. 1b).

There are some reviews for DIBs mainly focus on summarizing the advancements of cathode/anode materials and electrolytes, which have effectively promoted the development of the emerging battery systems. However, a systematic analysis of fundamental challenges arising from its specific working mechanism and corresponding strategies remains absent. In this review, we will first introduce the fundamental aspects of DIBs, including anion storage mechanism, capacity contribution mechanism, and latest design of electrode materials and electrolyte. We then focus on comprehensively discussing the challenges of DIBs, such as irreversible decomposition of electrolytes at high voltage, co-intercalation of solvent molecule, unsatisfactory electrolytes-electrodes compatibility in a wider voltage window, limited intercalation capacity, kinetics mismatch between cathode and anode, and excessive electrolyte. Following this, the corresponding strategies are discussed in detail, including designing high oxidation resistant and high electrochemical stable electrolytes, tailoring anion solvation structure, constructing sturdy electrode–electrolyte interphase, discovering cathode materials with high anion storage capability, overcoming safety issues at high operating voltage, and designing dual-carbon full-cells. Finally, we highlight the research prospects of advanced characterization technologies for components and structure investigation, theoretical calculations for probing anion intercalation chemistry, and AI for efficient discovery of electrode materials and electrolytes. We expect that this review can attract more attention and promote the practical applications of DIBs.

## Fundamental Understanding of DIBs

### Anion Intercalation Mechanism

During charge/discharge process, there are only Li^+^ shuttle between the cathode and anode in the conventional LIBs [23]. Taking LiCoO_2_ as an example, during charge, Li^+^ will be extracted from the cathode coupling with the oxidation of trivalent Co element. On the contrary, Li^+^ will be intercalated into the cathode, and the tetravalent Co element will undergo a reduction reaction. As for the DIBs, it is anions that can be reversibly (de)intercalated into the cathode materials, rather than Li^+^. Furthermore, traditional layered oxides struggle to maintain the framework integrity at the high voltage (> 4.8 V) that anion intercalation required. As a contrast, graphite exhibits excellent structural stability at this high voltage, enabling its reversible anions storage [24]. Graphite features a layered structure composed of stacked graphene planes stabilized by π-π interactions between *sp*^2^-hybridized carbon layers, with a hexagonal arrangement [23]. This unique architecture enables reversible electrochemical accommodation of diverse ionic species (including cations and anions), forming graphite intercalation compounds (GICs). The conjugated π-bonds in graphite effectively facilitate electron transfer, which makes it possible for accommodating cations at low potentials by forming donor-type GICs, while acceptor-type GICs formed at a high potential with the anion intercalation.

The formation process of GICs follows a “staging” mechanism, which relies on the balance between the van der Waals forces among the graphene layers and the repulsion of inserted ionic layers. The stage number (*n*) represents the number of graphene layers existing between two adjacent intercalant layers, which significantly impacts the intercalation capacity, and it can be calculated by the following equations using two characteristic (00*n* + 1) and (00*n* + 2) plane peaks in X-ray diffraction (XRD) patterns [19, 25, 26]:

1 $$d_{00n + 1} = I_{c} /(n + 1) = \lambda /(2\sin \theta_{00n + 1} )$$

2 $$d_{00n + 2} = I_{c} /(n + 2) = \lambda /(2\sin \theta_{00n + 2} )$$

3 $$n = [1/(\sin \theta_{00n + 2} /\sin \theta_{00n + 1} {-} 1)] {-} 1$$

4 $$I_{c} = d_{i} + (n {-} 1) \times 3.35 A^{ \circ } = \Delta d + n \times 3.35 A^{ \circ } = (n + 1) \times d_{00n + 1}$$

where n + 1 and *d*_00n+1_ represent the index of (00n + 1) planes and the distance between adjacent planes, respectively, *λ* is the x-ray wavelength, *I*_*c*_ represents the periodically repeating distance, *d*_*i*_ represents the intercalant gallery height, and Δ*d* is the gallery expansion.

In traditional LIBs, Li^+^ cations undergo different stages of intercalating into graphite anode, finally forming C_6_Li with a theoretical specific capacity of 372 mAh g^−1^, whereas anions will insert into the graphite cathode in the DIBs during the charging process following the formation of various acceptor-type GICs [27]. Typically, PF_6_^−^-based electrolytes have been widely used in DIBs, in which PF_6_^−^ anions will be inserted into graphite layers and form C_20_PF_6_ (stage Ⅰ) at full charge, corresponding to the theoretical specific capacity about of 112 mAh g^−1^ [28]. However, solvent molecules co-intercalating usually happens alongside anion intercalation at high voltage, making it difficult to achieve theoretical capacity. Furthermore, the geometries of different anions have significant influence on the structure stability and onset voltage. Typically, the large tetrahedral geometry of AlCl_4_^−^ will induce severe lattice strain and lattice distortion, resulting in irreversible graphite exfoliation despite its much lower onset voltage about ~ 2.0 V for intercalation [29]. Conversely, more symmetric octahedral PF_6_^−^ anion will induce more evenly distributed strain without obvious structural destroy and usually require higher intercalation voltages (> 4.3 V) [30]. The planar shape of TFSI^−^ will lead to a high in-plane packing density within the graphite gallery, which is facilitate to its high specific capacity of 140 mAh g^−1^ [31].

Cathode materials determine the electrochemical performance of DIBs by directly affecting the intercalation of anions [32, 33]. The increased graphitization degree of cathode materials can not only reduce the voltage hysteresis, but also improve the cycling stability and rate capability by enhancing electronic conductivity and reducing anion diffusion barriers. The electrochemical performance of graphite cathode for the anion reversible (de)intercalation is also influenced by its morphology and particle size [34–36]. Larger particle size can enhance the structural integrity and cycling lifespan but extend anion diffusion paths, consequently resulting in sluggish kinetics. Although this issue can be alleviated by designing graphite cathodes with higher specific surface area, severe side reactions would lead to aggravating challenges to structure stability. In addition, the intercalation of anions combined with the co-intercalation of solvent molecules at high voltage will induce severe volume expansion of over 130%, which would accelerate the graphite layers’ exfoliation and structure collapse [28].

### Anion Solvation Microenvironment

In traditional non-aqueous electrolytes of LIBs, the Li^+^ solvation structure is established through interactions between solvent molecules and ions, which critically governs the formation of the electrode–electrolyte interface and influences key parameters, such as Li^+^ transference number, diffusion coefficient, and electrochemical stability window [37, 38]. Therefore, understanding and tailoring the solvation structure is essential for optimizing electrochemical performance. In carbonate-based electrolytes, the cyclic carbonates with high dielectric constants exhibit strong Li⁺ coordination strength, preferentially occupying the inner solvation shell through cation-dipole interactions [39, 40]. Conversely, linear carbonates with lower dielectric constants are driven to the outer solvation sheath due to weaker electrostatic interactions. Although Li⁺ solvation structures have been extensively investigated, anion solvation structures, which play a critical determinant in DIBs, remain largely overlooked. This knowledge gap requires fundamental studies on anion diffusion in bulk electrolytes and desolvation at the electrode surface, as these processes critically affect reaction kinetics, oxidative stability, Coulomb efficiency, and service life of DIBs.

**Fig. 2:**
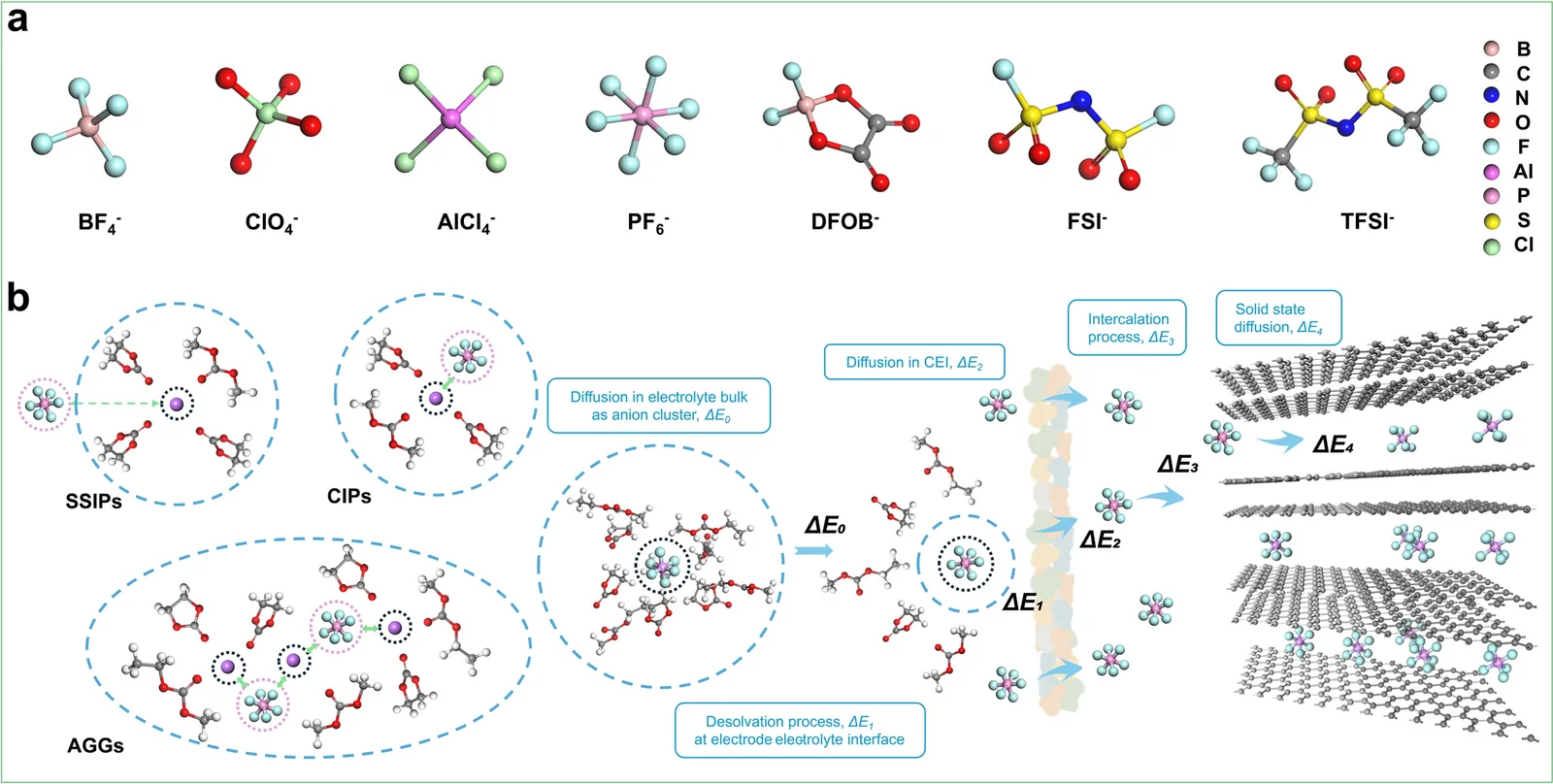
**a** Molecular structure of representative anions commonly employed in DIB electrolytes.** b** Schematic illustration of the solvation, diffusion, and desolvation processes of anions in bulk electrolytes

The performance of DIBs is governed by the storage capability of cathode materials, which is directly related to the physicochemical properties of anions, such as molecular size and structure, solubility in solvents, and electrochemical stability. Up to now, a series of anions including F^−^, Cl^−^, Br^−^, I^−^, PF_6_^−^, FSI^−^, TFSI^−^, BF_4_^−^, AlCl_4_^−^, ClO_4_^−^, and DFOB^−^ have achieved reversible (de)intercalation into graphite cathode (Fig. 2a) [24, 41, 42]. Except for simple halide ions, most anions consist of multiple atoms with halogen substituents surrounding a central atom, where the high electronegativity of fluorine or chlorine promotes charge delocalization. Unlike the localized point charge of Li^+^, this delocalization spreads the negative charge over a larger space, thereby diminishing electrostatic interactions [43–45].

Meanwhile, aprotic solvents in DIBs exhibit monopolar character with sterically shielded positive regions, which makes the solvent molecular less accessible to anions [46]. Suffering from this factor, anions usually performed relatively weaker interaction with solvents compared to cation-solvent coordination, consequently leading to a lack of well-defined solvation shells for anions. Such weakly solvated anions facilitate faster desolvation, endowing DIBs with the potential for rapid kinetics. Nevertheless, the anions intercalation chemistry is critically influenced by the anion surrounding microenvironment including the “solvation effect” of anion-solvent and anion-cation interactions.

As shown in Fig. 2b, during the charging process, similar with Li^+^ diffusion and intercalation process, anion transfer typically happens through sequential steps: first, anions diffuse in their solvated form through the bulk electrolyte; subsequently, the solvated anions undergo desolvation upon reaching the cathode surface; following this, bare anions will leave their solvated shell overcoming the desolvation energy (Δ*E*_1_) to pass through cathode-electrolyte interphase (CEI); finally, anions intercalate into graphite layers against the intercalation energy (Δ*E*_3_) and form GICs accompanied by electron loss [29, 37, 47–49]. The overall change in Gibbs free energy, determined by the combined effect of these two barriers, determines the intercalation potential (*φ*) during this process. Notably, the anion intercalation and GICs formation will be carried out efficiently when *φ* is within the oxidative limit potential of the electrolyte (*φ*_limit_), or the electrolyte will encounter irreversible decomposition.

However, the anions with small size and symmetric molecular structure will show strengthened anion-solvent interactions when dissolved in solvent with high polarity, which creates a higher energy barrier, inducing a higher onset intercalation potential. Due to the strong anion-solvent interaction, the phenomenon of solvent co-intercalation is inevitable during the anion intercalation process, leading to exfoliation and structural degradation of the graphite layers [50–53]. Thus, deliberate tailoring of the anion solvation microenvironment to promote the anion desolvation at the cathode surface and expanding the electrochemical window of electrolytes are key approaches to pursuing high-performance DIBs.

### Potential Window of DIBs

In LIBs, the battery voltage (V) is governed by the chemical potential difference of cathode and anode materials, as shown in Eq. (5) [54, 55]:

5 $$V = \Phi_{s,c} - \Phi_{s,a}$$

where $\Phi_{s,c}$ and $\Phi_{s,a}$ represent the potential of the cathode and anode, respectively. However, in DIBs, both the cations and the anions contributing to the capacity are provided by the electrolyte, whose concentrations in electrodes also influence the battery voltage.

The operating voltage of DIBs can be derived as in Eqs. (6–9). Using Nernst Eqs. (6 and 7), the chemical potential of Li^+^ ($\mu_{{Li^{ + } }}$) and A^−^ ($\mu_{{A^{ - } }}$) in the electrolyte can be evaluated, which show a close relationship with the concentration of cations and anions in the electrolyte. During the charging/discharging process, equivalent amounts (*n*) of anions and cations in the electrolyte will transfer toward opposite directions and inserted into electrodes. When the whole battery system is at its equilibrium state, the battery voltage (V) of DIBs can be expressed as Eq. (9) by combining Eqs. (6–8) [17].

6 $$\mu_{{Li^{ + } }} = \mu_{{Li^{ + } }}^{0} + kT\ln \left[ {Li^{ + } } \right]$$

7 $$\mu_{{A^{ - } }} = \mu_{{A^{ - } }}^{0} + kT\ln \left[ {A^{ - } } \right]$$

8 $$- neV = n(\mu_{Li} - \mu_{{Li^{ + } }} ) + n(\mu_{A} - \mu_{{A^{ - } }} )$$

9 $$- eV = \mu_{Li} + \mu_{A} - \mu_{{Li^{ + } }}^{0} - \mu_{{A^{ - } }}^{0} - kT\ln [Li^{ + } ] - kT\ln [A^{ - } ]$$

where A^−^ represents the anions, $\mu_{Li}$ and $\mu_{A}$ stand for the chemical potential of intercalated Li in the anode and A in cathode, respectively, while $\mu_{{Li^{ + } }}^{0}$ and $\mu_{{A^{ - } }}^{0}$ refer to chemical potential of Li^+^ and A^−^ in 1 mol L^−1^ solution under standard conditions with a pressure of 101.325 kPa and a temperature of 298 K, respectively.

From the above equations, it is clear that the voltage window of DIBs depends not only on the cathode and anode materials, but also on the types of cations and anions, as well as the salt concentration in the electrolyte. Although oxygen-free graphite cathode exhibits excellent oxidative resistance at a high intercalation voltage (> 4.8 V), the high working potential brings great challenges for designing the electrolyte systems. The electrolyte is required to provide an electrochemical stability window that can cover the operating voltage range of the DIBs [56–58]. The chemical potential of the anode must lie below the lowest unoccupied molecular orbital (LUMO) of electrolytes to impede electron transfer from the anode to the LUMO, preventing persistent electrolyte reduction on the anode surface. Meanwhile, the chemical potential of the cathode is demanded to be located above the highest occupied molecular orbital (HOMO) of electrolytes to avoid the electrolyte oxidation on the cathode surface induced by the electron transfer from the HOMO of the electrolyte to the cathode. Critically, the in situ formation of stable CEI and solid electrolyte interphase (SEI) during initial cycling provides kinetic stabilization, which enables operational voltage windows exceeding the thermodynamic stability limits of bulk electrolytes [59].

## Challenges of DIBs

**Fig. 3:**
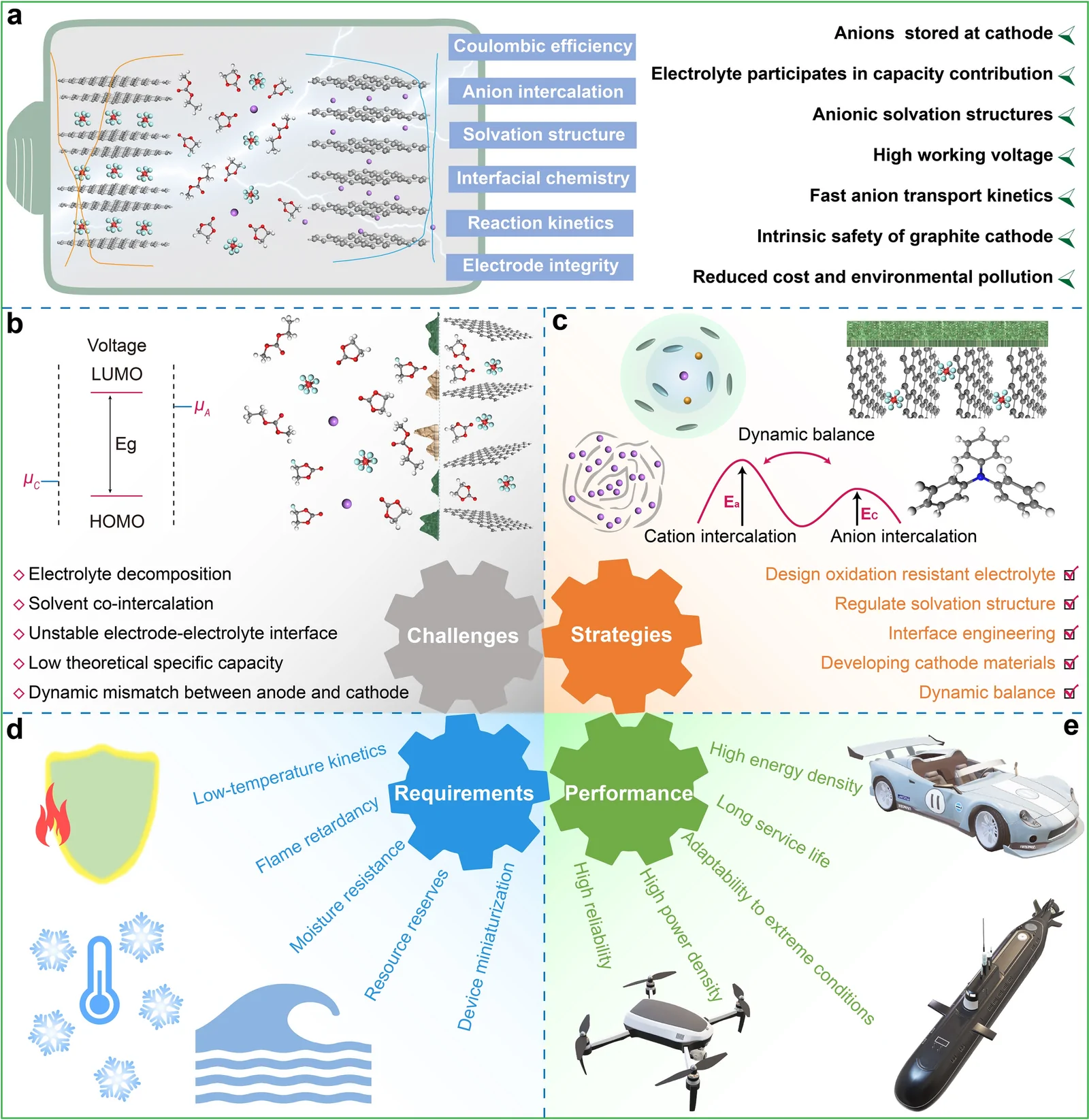
Comprehensive understanding of DIBs. **a** Advantages. **b** Challenges. **c** Strategies. **d** Requirements. **e** Performance

As an emerging next-generation battery system, DIBs have attracted significant attention due to their promising potential to simultaneously integrate high energy density and high-power density, offering broad prospects for commercial applications. As shown in Fig. 3a, based on a unique anion intercalation mechanism, DIBs provide high operating voltages, intrinsic electrode safety, fast reaction kinetics, and potential cost advantages from Earth’s abundant electrodes. However, the distinctive mechanism brings specific challenges including high-voltage electrolyte decomposition, solvent co-intercalation, unstable electrode–electrolyte interface, limited capacity, and dynamic mismatch between the cathode and the anode (Fig. 3b). To address these issues, as shown in Fig. 3c, different strategies have been developed such as designing oxidation resistant electrolytes, regulating anion solvation microenvironment, constructing robust interface, discovering new materials, and balancing dynamic between both electrodes. Based on these approaches, DIBs are expected to become a promising secondary battery system with advantages including high energy density, high power density, long service life, high safety, as well as the adaptability to extreme conditions (Fig. 3d, e). In this section, the challenges of DIBs will be systematically summarized and discussed in detail.

### Electrolyte Decomposition Induced by High Operating Voltage

Driven by the anion intercalation mechanism, DIBs typically operate at voltages significantly higher than those of conventional LIBs, therefore impose stringent requirements on electrolyte stability. Most conventional organic solvents, including carbonate ester and ether, are highly susceptible to oxidative decomposition under the elevated cathode potentials, leading to gaseous products, such as carbon oxides, olefins, and acidic byproducts (e.g., HF in the presence of fluorinated species), and a variety of organic and inorganic degradation compounds [37, 38]. Additionally, anions found in widely used lithium, sodium, and potassium salts such as PF_6_^−^, TFSI^−^, and FSI^−^ are also prone to oxidative decomposition at high potentials [60]. Furthermore, owing to the catalytic nature of graphite defects and edge sites, electrolyte stability in graphite-based DIBs extends beyond solvent-intrinsic thermodynamic descriptors (e.g., HOMO levels), particularly at voltages > 4.8 V. In contrast to the inert basal planes (002), edge planes and surface defects exhibit significantly higher electronic density of states. These active sites facilitate substantially faster electron transfer kinetics and stronger chemical adsorption, thereby accelerating electrolyte oxidation well before the bulk solvent reaches its theoretical voltage limit [61].

Electrolyte decomposition under high-voltage conditions not only compromises the integrity of the electrolyte itself but also triggers a cascade of harmful effects that severely impact battery performance and safety. These effects can be understood from two interwoven perspectives: the irreversible loss of functional electrolyte components, and the detrimental consequences of the decomposition byproducts. First, the oxidative breakdown of anions and solvents is often accompanied by consumption of alkali metal cations, producing stable salts such as LiF and NaF [62, 63]. This irreversible ion loss depletes the reservoir of active charge carriers and leads to continuous capacity fading. Concurrently, solvent degradation reduces the effective electrolyte volume and increases viscosity, which collectively deteriorates ion transport and overall electrochemical performance [64]. Second, the decomposition products themselves introduce severe secondary hazards. Reactive intermediates such as PF_5_, free radicals, and HF act as chemical catalysts that accelerate further decomposition of other components through chain and cross-reactions [65]. For instance, PF_5_, generated from LiPF_6_, acts as a strong Lewis acid, catalyzing solvent breakdown, while solvent-derived radicals attack anions or neighboring solvent molecules. Quantitative analysis using ^19^F NMR, spectroscopic ellipsometry and on-line electrochemical mass spectrometry (OEMS) has confirmed that these parasitic reactions can rapidly accumulate HF and gaseous POF_3_ concentrations to 50–100 ppm [66–68]. Furthermore, Differential Electrochemical Mass Spectrometry (DEMS) has been extensively utilized in recent years as a high-precision analytical tool [69]. It enables the quantitative real-time monitoring of HF evolution, alongside H_2_ and other volatile byproducts, providing critical insights into the degradation kinetics. This highly corrosive HF damages both electrode materials and current collectors, leading to metal dissolution and contact failure [70]. These processes destabilize the electrode–electrolyte interphases. On the cathode side, the decomposition products easily lead to the formation of thick, porous, and ionically resistive CEI layers that hinder anion intercalation. On the anode side, migrated oxidative and acidic species poison the SEI and induce dendrite formation and dead metal accumulation [71, 72]. Furthermore, gaseous byproducts such as CO_2_, CO, H_2_, hydrocarbons, and SO_2_ contribute to cell swelling, internal pressure build-up, and serious safety risks including rupture and thermal runaway [73]. These issues make it difficult to maintain high Coulombic efficiency in DIBs and keep their cycle life at relatively low levels. Therefore, developing new electrolyte systems that can operate stably at high voltages has consistently been a key challenge in DIBs research.

### Co-Intercalation of Solvent Molecules

During the charging process of DIBs, anions such as PF_6_⁻ are ideally expected to intercalate into the cathode host, such as graphite, driven by the strong Coulombic force under high voltage [52, 74, 75]. However, in practical polar solvent-based electrolytes, anions are typically solvated and often exist in ion cluster form. When the desolvation barrier is prohibitively high, entire solvated anion-solvent complexes may be pulled into the cathode interlayers, leading to the solvent co-intercalation [76]. This effect is further amplified in systems involving highly polar solvents and bulky anions. Highly polar solvent molecules experience strong Coulombic attraction from the positively charged electrode under high voltage, making them prone to direct intercalation. At the same time, bulky anions tend to form large solvation complexes with multiple coordination sites, increasing the likelihood that entire anion–solvent clusters are pulled into the graphite interlayers. These combined factors significantly enhance the probability of solvent co-intercalation. The most significant and direct harm of solvent co-intercalation is the reduction of reversible electrode capacity by occupying sites originally intended for anion storage. Additionally, co-intercalation during the initial intercalation stages may cause unpredictable expansion and collapse of cathode material’s structure [53]. These irreversible phase transitions block the migration of already intercalated anions and prevent new intercalation. Moreover, once co-intercalated solvents undergo decomposition under high voltage, additional gas and solid byproducts may further aggravate structural instability and transport resistance. Therefore, the solvent co-intercalation issue is an urgent challenge to be addressed in DIBs.

### Instability of the Electrode–Electrolyte Interphase

The electrode–electrolyte interface consists of the CEI on the cathode and SEI on the anode. In DIBs, CEI faces more demanding requirements: it must both block solvent decomposition for electronic isolation and facilitate the transport of larger anions for efficient ionic conduction. CEI formation is therefore not a simple passive outcome of electrolyte decomposition but is strongly coupled with complex electrochemical and chemical processes such as charge transfer during anion intercalation and destruction of solvation sheaths, making CEI research particularly challenging [77, 78]. Due to its violent formation process under high potential, the CEI layer often grows into a thick, porous, and mechanically fragile layer. In contrast to LIB cathodes that typically undergo ~ 10% volume change, DIB cathodes may experience > 100% expansion/contraction during anion co-intercalation, imposing stringent mechanical requirements on the CEI [79]. It must remain mechanically compliant yet damage-tolerant to accommodate repeated strain without cracking or delamination, enabled by adequate elasticity, high fracture resistance, and strong interfacial adhesion. More importantly, oxidative decomposition products generated at high cathode potentials, such as active oxygen and acidic substances like HF, may migrate to the anode to attack and damage the already fragile SEI, triggering continuous side reactions and dendrite growth [80, 81]. Reductive species from anode may migrate to the cathode may be oxidized at the high cathode potential, further exacerbating the interface side reactions and CEI instability. This “cross-contamination” is severe in DIBs with wide voltage windows and intense interfacial reactions, forming a vicious cycle. The interfacial phase problem presents a challenge to the entire battery system. It requires high-voltage electrolyte design and functional additive development, as well as exploration of electrode material surface modification and coating, while optimizing battery operating conditions. A multi-pronged approach is needed to address the stability issues of both cathode CEI and anode SEI under extreme potential.

### Asymmetric Reaction Kinetics of Cathode and Anode

In DIBs, a significant kinetic asymmetry exists between the cathode and anode reactions. At the cathode, anion intercalation into graphite-like layered materials occurs under high voltage, where the strong electrochemical driving force facilitates rapid charge transfer. Despite the large ionic radius of anions such as PF_6_^−^ and TFSI^−^, the low migration barriers within the cathode overcome traditional bottlenecks of solid-phase transport. This enables the cathode to sustain high-rate operation with relatively low polarization, making it well-suited for high-power applications [12, 13, 15]. In contrast, anodic reaction is governed by sequential steps, including desolvation of solvated metal ions, charge transfer across the electrode/electrolyte interface, and solid-state diffusion in the anode. Among them, desolvation plays a critical role, especially under high current conditions, as alkali metal ions in polar electrolytes are often strongly coordinated with solvent molecules, and energy barrier for shedding the solvation shell can become a kinetic bottleneck [82, 83]. Beyond desolvation, the transport kinetics of alkali metal ions across the SEI layer are of paramount importance. During high-rate charging and discharging, the ionic transport resistance surges within the SEI layer, becoming a critical kinetic impediment. Furthermore, charge transfer kinetics at the electrode/electrolyte interface can be sluggish, hindering the rapid exchange of electrons and ions. For graphite anodes, solid-state diffusion of active ions within the anode material can also become a rate-limiting factor, particularly in thicker electrodes or at high current densities, restricting fast ion intercalation or deintercalation.

The anode inability to kinetically match the cathode fast reaction rates leads to several significant performance issues. These include sharp voltage fluctuations, premature cutoffs during charging or discharging, and plunged Coulombic efficiency. Furthermore, the uneven deposition kinetics, exacerbated by the factors mentioned above, often induce the formation of dendrites on the alkali metal anode surface. Addressing this kinetic asymmetry is essential to realizing the full power potential of DIBs. This necessitates a multifaceted strategy, with particular emphasis on the development of high-voltage-stable electrolytes and advanced interfacial engineering approaches.

### Limited Capacity and Energy Density

The limited theoretical specific capacity and energy density of DIBs are predominantly determined by a fundamental constraint in the cathode material for ion storage, particularly for anions. The anion accommodation capability of graphite cathode is restricted by its rigid layered structure with a narrow interlayer spacing (~ 0.335 nm), which is unfavorable for reversible (de)intercalation of polyatomic anions with a large diameter (e.g., PF_6_^−^: 0.35 nm, TFSI^−^: 0.80 nm) [84–87]. As a result, it is difficult for the graphite to deliver a theoretical capacity exceeding 140 mAh g^−1^ even at a deep charge state with the formation of C_20_PF_6_ (stage Ⅰ). Although expanding the interlayer spacing can accommodate anions with large sizes, the structural integrity of graphite is compromised during the extended cycling test. Meanwhile, the high operating voltage will induce inevitable electrolyte oxidation and form resistive interphases at the cathode surface that hinders the anion diffusion and intercalation, which will further diminish the practical capacities. In addition, graphite suffers from inherently low tap density compared to that of commercial cathode materials for LIBs, such as nickel-rich layered oxides, and LiCoO_2_. To achieve the same capacity load, the graphite cathode must be designed with greater thickness due to its volumetric disadvantage, at the cost of reducing the energy density of the full cell. To address this issue, researchers need not only to develop new anion storage materials with higher theoretical capacity, but also to combine morphology and structural engineering to improve the electrochemical stability and further enhance their energy density.

Moreover, the anions in the electrolyte will undergo reversible (de)intercalation at the graphite cathode in DIBs, indicating that the electrolyte itself functions as an active material, which directly contributes to the capacity. Compared to rocking-chair batteries, this anion intercalation mechanism makes a distinct requirement for larger quantity of electrolyte, which is not only consumed to form stable SEI but also experiences concentration fluctuations during continuous charging and discharging. For example, when a 1 M dilute electrolyte is used, its mass can account for over 70% of the total battery weight, which severely limits the practical energy density of DIBs (often below 50 Wh kg^−1^) [12, 88]. Increasing the salt concentration while reducing the proportion of inert solvent is a promising strategy to address this issue, thereby enhancing the energy density of the DIBs system. Pursuing this approach to its limit, the development of solvent-free electrolytes can effectively improve their energy density with a high theoretical value of 246 Wh kg^−1^ and projected cell-level energy density reaching 172 Wh kg^−1^ [89].

### Reduced Capacity under Low Temperatures

The electrochemical reaction kinetics of batteries are highly temperature-dependent. As the temperature decreases, solvent molecules demonstrate higher dielectric constants and stronger polarity, which intensify solvent–solvent interaction and viscosity, ultimately approaching the freezing point [90, 91]. Meanwhile, enhanced ion–solvent and ion-ion interactions promote the formation of large solvated ion clusters through tight coordination between solvents, anions, and cations, therefore inevitably impeding the ion transport in bulk electrolytes [92, 93]. The solvents will show sharply reduced salt solubility, precipitating salt crystals at low temperatures and further diminishing the concentration of charge-carrier ions in the electrolyte. As a result, both cations and anions exhibit sluggish diffusion kinetics in the bulk electrolyte as the temperature decreases. Furthermore, the increased ion–solvent and cation–anion interactions within the solvation sheath will enhance the desolvation energy barriers of both cations and anions at the surface of SEI and CEI, respectively. Following desolvation, the ions will diffuse across the thickened SEI/CEI films, exhibiting higher impedance compared to that at room temperature and promoting metallic plating and dendrites formation at the anode side. These factors consequently exacerbate the polarization of DIBs, increasing charge/discharge overpotential, and aggravating capacity fade with reduced rate capacity. Therefore, it is necessary to design the solvation structure and the electrode–electrolyte interface chemistry to maintain excellent cation/anion transport kinetics in low temperature, ensuring the cycling stability and rate performance of DIBs.

### Safety Issues

The serious safety concern in rechargeable batteries stems from thermal runaway, which is usually induced by internal defects like lithium dendrites or separator flaws or external abuse including mechanical crush and overcharging [29]. The main cause of safety issues is exothermic chemical reactions within the batteries, which originate from the release of reactive oxygen species (O*) from delithiated layered oxide cathodes and flammable organic liquid electrolytes. Although DIBs do not use oxygen-containing cathode materials, they still use organic electrolytes suffering from irreversible decomposition, generating large amounts of gas and oxygen-sensitive organic byproducts. Subsequently, the continuous rise in battery temperature and internal pressure further accelerates harmful side reactions, igniting combustible electrolyte vapors, and even leading to battery explosion. Notably, each stage and temperature change of thermal runaway are influenced by factors such as the type of electrode materials as well as their morphology and structure, and the physicochemical properties of electrolytes and separators. Of course, the runaway process remains dominated by electrochemical reactions, such as the oxidation of flammable electrolytes, and the formation of dendrites, exceeding the tolerance of the cell system. Numerous studies have revealed that designing novel electrolytes with excellent oxidative resistance and flame retardancy can alleviate this problem. Meanwhile, strategies for designing separators with excellent mechanical strength and flame retardancy are also a feasible approach to improve battery reliability.

### Self-Discharge at High Voltage

Unlike conventional LIBs, high-voltage DIBs notoriously suffer from severe self-discharge, a critical bottleneck that restricts their shelf life and practical deployment. The genesis of this phenomenon lies in a synergistic interplay between thermodynamic instability and intrinsic structural metastability, driven by extreme operating potential.

The primary reason for happening involves complex coupled mechanisms, where physical structural instability exacerbates chemical decomposition. From a microscopic physical perspective, the intrinsic metastability of GICs plays a pivotal role. First-principles calculations have theoretically revealed that reaching the fully intercalated Stage-I phase induces significant Coulombic repulsion between adjacent anionic layers and causes a massive lattice expansion (e.g., ~ 114% for PF_6_). This creates a high-energy thermodynamic state characterized by severe mechanical strain [94]. Consequently, anions tend to spontaneously de-intercalate from the graphite lattice during open-circuit rest to relax this structural stress. This theoretical prediction is corroborated by in situ Raman spectro-electrochemistry studies, which visually tracked the spontaneous reverse phase transition of FSI-GICs (from Stage-II to dilute stages) during rest, confirming that anion loss is a primary driver of the rapid voltage decay [40].

Simultaneously, severe self-discharge challenges also stem from the anode side. Due to insufficient binding interaction between the host material and the inserted species, active cations tend to spontaneously detach from the anode structure and dissolve back into the electrolyte during static storage [95]. This spontaneous release of charge carriers directly accelerates voltage decay and capacity fading, necessitating anode designs with enhanced ion-binding capabilities to stabilize the charged state. Enhancing the oxidative stability of electrolytes is inherently a system-level endeavor, it is governed not only by the intrinsic properties of the electrolyte components but also by the interactions and coupling among the other cell components.

## Strategies for DIBs

As discussed in Sect. 3, critical issues remained for DIBs, such as electrolyte decomposition at high work voltage, solvent molecules co-intercalation, unstable electrode–electrolyte interface, dynamic mismatch between cathode and anode, limited capacity and energy density, poor electrochemical performance under low temperatures, and safety issues. To address the problems mentioned above and fully employ the advantages of DIBs, it is urgent to develop different strategies that involve cathode and anode materials, electrolyte systems, and separators. In this section, we will discuss attempts towards constructing high-performance DIBs.

### Strategies for High Voltage Electrolyte

Overcoming the challenges associated with high-voltage operation in DIBs requires a comprehensive approach to electrolyte design. Specifically, electrolyte components must simultaneously exhibit oxidative stability, support effective ion transport, and enable the formation of robust interfacial phases. The molecular engineering of high-voltage solvents, control over solvation environments, and rational additive design represent the three major research directions for achieving robust high-voltage electrolyte systems. The physical properties of different solvent molecules are compared in Table 1.

**Table 1:** Comparison of physical properties of different solvent molecules

Solvent	Molecular formulae	Melting point (°C)	Boiling point (°C)	Viscosity at 25 °C (cP)	Permittivity (ε) at 25 °C	HOMO (eV)	LUMO (eV)	Refs
Ethylene carbonate (EC)	C_3_H_4_O_3_	35	244	2.00	89.00	− 8.47	− 0.28	[96–98]
Dimethyl carbonate (DMC)	C_3_H_6_O_3_	3.00	90.00	0.70	3.00	− 8.22	0.08	[96, 97]
Ethyl methyl carbonate (EMC)	C_4_H_8_O_3_	− 53.00	110.00	0.65	2.90	− 8.13	0.07	[96, 97]
Propylene carbonate (PC)	C_4_H_6_O_3_	− 48.80	242.00	2.53	64.90	− 8.37	− 0.30	[96, 97]
Diethyl carbonate (DEC)	C_5_H_10_O_3_	− 43.00	127.00	0.80	3.00	− 8.05	0.07	[96, 99]
Vinylene Carbonate (VC)	C_3_H_2_O_3_	22.00	162.00	1.54	126.00	− 7.37	− 0.57	[96, 100]
1,2 -Butylene carbonate (BC)	C_5_H_8_O_3_	− 53.00	240.00	3.12	53.00	− 8.33	− 0.34	[101, 102]
Fluoroethylene carbonate (FEC)	C_3_H_3_O_3_F	17.30	210.00	4.10	67.00	− 8.97	− 0.39	[96, 100]
1,2-Dimethoxy ethane (DME)	C_4_H_10_O_2_	− 58.00	84.00	0.46	7.20	− 7.19	0.23	[96, 103]
Tetrahydrofuran (THF)	C_4_H_8_O	− 109.00	66.00	0.46	7.40	− 7.09	0.16	[104]
Dioxolane (1,3-dioxolane (DOL))	C_3_H_8_O_2_	− 95.00	78.00	0.59	7.10	− 7.36	0.12	[104, 105]
Ethyl acetate (EA)	C_4_H_8_O_2_	− 84.00	77.00	0.45	6.02	− 7.70	− 0.02	[96, 104]
Methyl acetate (MA)	C_3_H_6_O_2_	− 98.20	57.00	0.37	6.70	− 7.79	− 0.08	[96, 104]
1,2-Diethoxyethane (DEE)	C_6_H_14_O_2_	− 74.00	121.00	0.56	5.10	− 7.11	0.22	[96, 104]
Dimethyl formamide (DMF)	C_3_H_7_ON	− 61.00	153.00	0.80	37.00	− 6.97	− 0.15	[101]
Tetramethylene sulfone (TMS)	C_4_H_8_O_2_S	28.00	287.00	10.00	43.00	− 7.88	− 0.36	[101, 102]
Dimethyl sulfoxide (DMSO)	C_2_H_6_OS	19.00	189.00	1.37	47.00	− 6.49	− 0.15	[96, 101, 106]
Ethylene sulfite (ES)	C_2_H_4_O_3_S	− 17.00	174.00	2.06	39.60	− 8.21	− 1.26	[96, 107]
Trimethyl phosphate (TMP)	C_3_H_9_O_4_P	− 46.00	197.00	2.20	21.00	− 8.11	− 0.09	[100, 101]
Triethyl phosphate (TEP)	C_6_H_15_O_4_P	− 56.00	215.00	13.10	1.60	− 7.95	− 0.10	[100, 108]
Dimethyl methyl phosphonate (DMMP)	C_3_H_9_O_3_P	− 50.00	181.00	1.75	22.30	− 7.90	− 0.12	[109, 110]
1-Methyl-2-pyrrolidinone (NMP)	C_5_H_9_ON	− 24.00	204.00	/	/	− 6.74	− 0.08	[102]
4-Trifluoromethyl-1,3-dioxolan-2-one (TFPC)	C_4_H_3_O_3_F_3_	− 4.00	~ 85.50	5.00	67.00	− 8.97	− 0.39	[102, 106]
Methyl 2,2,2-trifluoroethyl carbonate (TFEMC)	C_4_H_5_F_3_O_3_	− 44.00	90.00	1.00	9.60	− 8.76	− 0.16	[96, 102]
Methyl butyrate (MB)	C_5_H_10_O_2_	− 84.00	102.00	0.60	5.50	− 7.74	− 0.01	[96, 104]
Ethyl butyrate (EB)	C_6_H_12_O_2_	− 93.00	120.00	0.71	5.10	− 7.65	0.04	[96, 104]
Dimethoxymethane (DMM)	C_3_H_8_O_2_	− 105.00	41.00	0.33	2.70	− 7.17	0.06	[96, 104, 105]
Adiponitrile (ADN)	C_6_H_8_N_2_	1.00	295.00	6.10	30.00	− 9.28	− 0.59	[97]

**Fig. 4:**
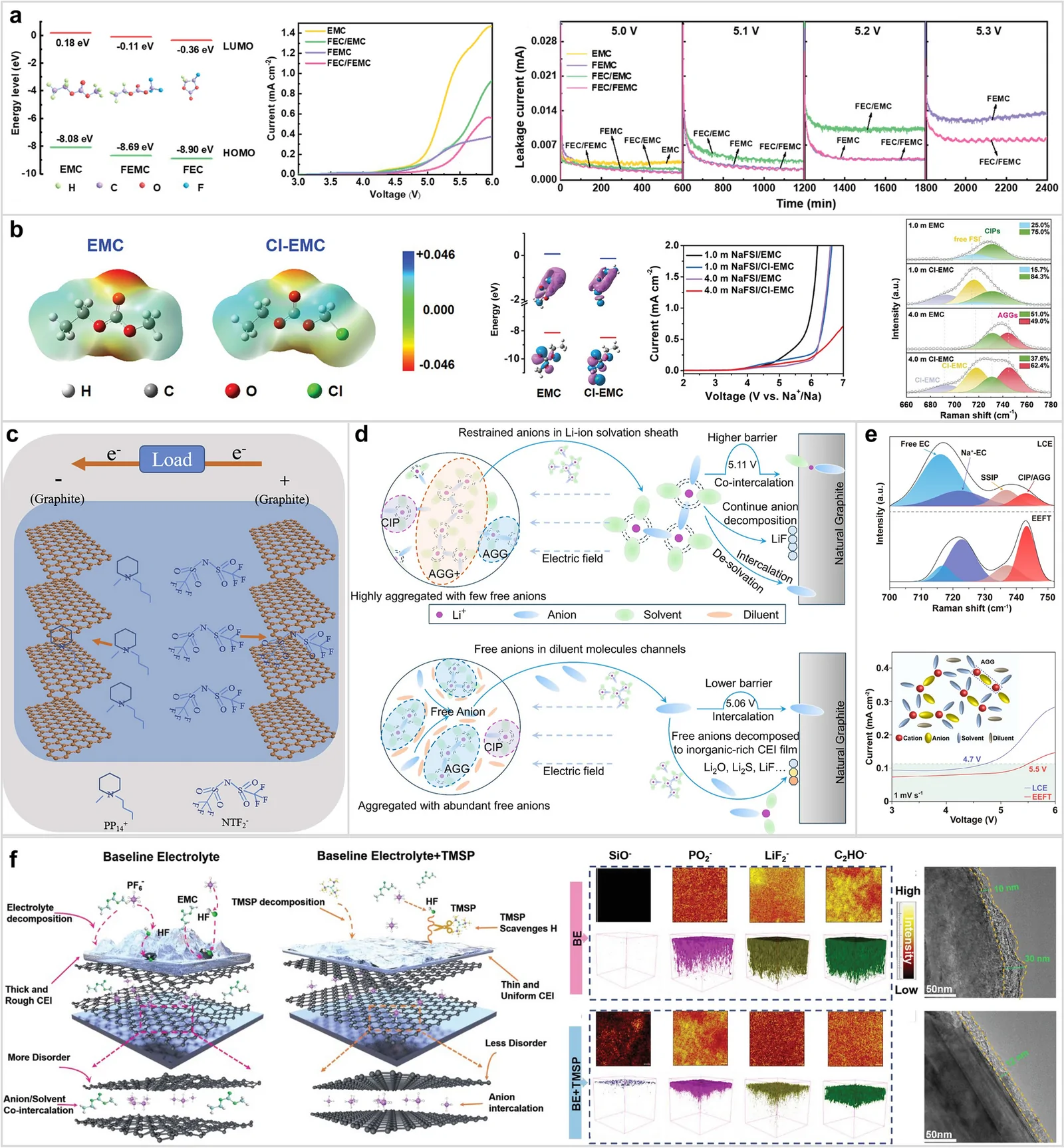
**a** Structures and molecular orbital energies of EMC, FEMC, and FEC solvents; LSV of a titanium electrode in 1 M LiPF_6_-based EMC, FEC/EMC, FEMC, and FEC/FEMC electrolytes; Potentiostatic profiles of graphite||Li cells with various electrolytes [115]. Copyright 2022, Wiley–VCH. **b** Comparison of molecular structure and ESP of EMC and Cl-EMC; Energy level comparison of EMC and Cl-EMC; Oxidative stability comparison of different electrolytes (scanning rate: 1 mV s^−1^); Raman spectra comparison of 1.0 and 4.0 mol L^−1^ electrolytes with EMC and Cl-EMC solvents [116]. Copyright 2020, Wiley–VCH. **c** Schematic illustration of the charging mechanism of the GGDIB based on PP_14_NTF_2_ ionic liquid electrolyte [124]. Copyright 2018, Elsevier. **d** Illustration of highly aggregated ion pairs with few free anions and anion behaviors in HCE and LHCE [136]. Copyright 2025, America Chemical Society. **e** Raman spectrum, LSV curves of LCE and EEFT electrolytes [50]. Copyright 2025, Wiley–VCH. **f** Schematic representation showing the problems induced by the unique functions of TMSP additive; TOF–SIMS investigations and HRTEM images of the graphite cathodes after 100 cycles of basic electrolyte (BE), and TMSP in BE [138]. Copyright 2022, Wiley–VCH

Enhancing solvent oxidative stability through molecular engineering is a primary strategy for developing high-voltage electrolytes. Fluorination is among the most widely adopted strategies. Owing to high electronegativity and strong electron-withdrawing nature, the introduction of fluorine atoms into solvent molecules lowers the electron density around oxidation-prone sites and stabilizes the molecular orbitals [111–113]. This results in a lower HOMO energy level, reducing the likelihood of electron extraction under high potential and thereby suppressing oxidative decomposition at the cathode [37]. Fluorinated carbonate solvents, such as fluoroethylene carbonate (FEC) and fluorinated ethyl methyl carbonate (F-EMC), have been widely studied and shown to significantly extend electrolyte voltage windows. As early as 2014, Read et al. designed a high-voltage electrolyte based on fluorinated versions of common carbonate solvents and additive by dissolving LiPF_6_ in FEC: EMC (4:6 w/w) + 5 mM tris(hexafluoro-iso-propyl) phosphate (HFIP) [114]. This electrolyte enabled PF_6_^−^ intercalation into graphite at 5.2 V with a capacity of 80 mAh g^−1^. The high-voltage stability of the electrolyte allowed full graphite cells to cycle for 50 cycles before their capacity dropped below 70% of the initial value. An all-fluorinated electrolyte system composed of 1 mol L^−1^ LiPF_6_ in a FEC/FEMC (vol:vol = 3:7) solvent mixture was designed to enable high-voltage DIBs with graphite cathodes operating up to 5.2 V [115]. As evidenced by theoretical calculation for molecular orbital, linear sweep voltammetry (LSV) and potentiostatic tests in Fig. 4a, the fluorinated carbonates exhibit reduced oxidative reactivity, contributing to remarkable interfacial stability. The resulting system maintains 94.5% capacity retention after 5000 cycles and delivers excellent high-rate capability with 91.8% capacity utilization at 50 C. Chlorination design can achieve similar effects to fluorination. As shown in Fig. 4b, by employing chloromethyl ethyl carbonate (Cl-EMC) as the solvent, the chlorination-based electrolyte showed a milder effect on solvent polarity and coordination structure due to weaker electronegativity of the introduced chlorine [116]. This also causes the HOMO and LUMO orbital energy level of the molecule to shift downwards and solvation structure to emerge more ion pairs, which enhances its antioxidant performance, as further confirmed by the LSV results and the Raman results. Utilizing a 4.0 mol L^−1^ NaFSI/Cl-EMC electrolyte, the resulting Na||graphite sodium-based DIBs delivered a reversible capacity of 104.6 mAh g^−1^ with negligible capacity fade over 900 cycles.

Functional group modification is another strategy to enhance solvent oxidative stability. Sulfone-based compounds and ionic liquids have also attracted considerable attention for their intrinsic oxidative stability [117, 118]. Sulfone groups (–SO_2_–) feature strong electron-withdrawing capability and high polarity, which contribute to improved anodic stability and electrochemical inertness [119]. A 5.2 mol L^−1^ KFSI/tetramethylene sulfone (TMS) concentrated electrolyte was developed for high-performance potassium-based DIBs, which exhibits a high oxidation potential due to TMS molecules getting close to the anions, enhancing the intercalation reversibility of FSI⁻ and improving K⁺ storage at the graphite anode [120]. Meanwhile, ionic liquids, composed of bulky organic cations and stable anions, exhibit wide electrochemical windows, low vapor pressure, and excellent thermal stability, making them suitable for co-solvents or additives under high-voltage conditions [121–123]. These classes of compounds offer complementary design strategies to high-voltage solvents for achieving robust electrolyte formulations. For instance, a novel DIBs using the ionic liquid electrolyte N-butyl-N-methyl-piperidinium bis(trifluoromethyl sulfonyl)imide(PP_14_NTF_2_) was designed by Li et al. displayed in Fig. 4c, demonstrating excellent charge–discharge performance, high capacity, and impressive cycle stability [124]. The use of ionic liquid enables high ionic conductivity, allowing the battery to operate effectively over a wide voltage window, with minimal capacity loss after 100 cycles. Among these, nitrile groups (− CN) are particularly promising due to their strong electron-withdrawing nature and multifunctional chemical behavior and have been widely applied in high-voltage electrolyte systems for LIBs. Incorporating nitrile functionalities can significantly enhance oxidative resistance. The − CN group stabilizes molecular orbitals by lowering electron density on oxidation-prone sites and acts as a strong proton acceptor and inhibiting reactive intermediates like free radicals during high-voltage operation. Guo et al. introduced 1,4-dicyanobenzene (DCB) as a nitrile additive in a pentaerythritol tetraacrylate-based gel polymer electrolyte (GPE) for high-voltage lithium metal batteries [125]. The nitrile additive modifies the solvation structure of Li⁺, creating a pseudo-concentrated electrolyte with improved electrochemical stability and enhanced compatibility with both the lithium metal anode and high-voltage cathode.

In addition to halogenation and above functional group modification, methylation and carbon chain extension are also useful for enhancing solvent oxidative stability, which have been often applied in conventional LIBs [126]. Introducing methyl groups in place of terminal hydrogen atoms reduces the electron-donating capacity of the molecule and lowers the HOMO energy level, making it less prone to oxidation under high voltage [127, 128]. Moreover, extending the alkyl chain length increases the molecular size and disperses the electron density, further stabilizing the molecular orbitals. Long-chain alkyl solvents tend to exhibit improved oxidative resistance due to reduced localized reactivity and enhanced steric hindrance, which suppresses direct interactions with the cathode surface. These structural modifications collectively strengthen the molecular robustness of solvents against oxidative decomposition [129, 130].

Tuning the solvation structure of electrolyte components offers an effective route to improving oxidative stability in high-voltage systems. In conventional electrolytes, solvation typically occurs in the form of solvent-separated ion pairs (SSIPs), CIPs, AGGs, and free solvent molecules [131, 132]. Among these, free solvents, particularly those uncoordinated with any salt ions, are the most vulnerable to oxidative decomposition, as their HOMO levels remain high and unshielded [133]. In contrast, coordinated solvent molecules involved in CIP, SSIP, or AGG structures generally exhibit lowered HOMO energy levels due to strong electrostatic interactions with the cation metal ions, which stabilize their electron clouds and reduce their oxidative activity. This fundamental understanding provides a clear rationale for enhancing electrolyte voltage tolerance by modulating the local solvation environment [134]. Localized high-concentration electrolytes (LHCEs) system embody this strategy by intentionally increasing salt-to-solvent ratios while incorporating inert, non-coordinating diluents to maintain acceptable fluidity. In LHCE systems, most solvent molecules participate in ion solvation, significantly reducing the population of free solvent species. The addition of highly fluorinated ethers, such as 1,1,2,2-tetrafluoroethyl-2,2,3,3-tetrafluoropropyl ether (TTE), serves two purposes: these molecules are chemically resistant to oxidation due to their low HOMO levels, and they possess extremely weak donor capability, rendering them non-coordinating with alkali cations [133, 135]. As a result, the solvation structure in LHCEs is enriched with contact ion pairs and aggregates, where anions increasingly occupy the primary solvation shell. This promotes anion-derived decomposition at the electrode interface, favoring the formation of inorganic species such as LiF, which contributes to the construction of compact, ionically conductive, and chemically robust interphases.

For instance, Hu et al. designed an LHCE based on TTE and TMS solvents, which enhances anion redistribution and stabilizes the electrolyte at high voltage as shown in Fig. 4d [136]. By facilitating a higher proportion of CIPs and AGGs in solvation structure, the LHCE promotes the formation of a robust, inorganic-rich CEI layer. This enhanced interfacial chemistry suppresses dendrite growth and significantly boosts cycling stability, with the battery demonstrating an impressive 2400 cycles at 85% capacity retention, a marked improvement over conventional high or low concentration electrolytes. Moreover, a LHCE was designed via dissolving LiFSI in tetramethylene sulfone (SUL) with TTE as a diluent to optimize the solvation structure and enhancing ion diffusion at high voltage [137]. This electrolyte forms a stable electrode–electrolyte interface with abundant inorganic fluorides, enabling durable anion intercalation and long-term cycling beyond 3000 cycles at 5.2 V. Additionally, its low viscosity and excellent wettability reduce electrolyte usage, showing promising potential for high energy density and practical applications. In addition, 3 mol L^−1^ NaPF_6_ dissolved in a mixture of EC, EMC, and FEC with TTE as a diluent, named EEFT, was developed as LHCE for sodium-based DIBs [50]. The Raman results in Fig. 4e reveal that the EEFT electrolyte’s optimized solvation structure promotes a higher proportion of ion pairs, such as stable CIPs and AGGs. This modification in solvation enhances the CEI stability and improves the oxidation resistance at high voltage, with the latter being confirmed by the LSV results. These synergistic effects enable the EEFT electrolyte to maintain low viscosity and high ionic conductivity while preventing solvent co-intercalation, leading to excellent rate capability (97.4 mAh g^−1^ at 100 C) and outstanding cycle stability (81% capacity retention after 10,000 cycles). This solvation-level control achieved through the synergistic design of salt concentration and diluent identity, offers a powerful and flexible strategy to push the oxidative voltage limits of electrolytes.

The incorporation of functional additives plays a pivotal role in further stabilizing high-voltage electrolyte systems. Sacrificial film-forming additives, especially those targeting cathode surfaces, can decompose preferentially to form protective interphase layers, mitigating continuous electrolyte degradation [139]. Examples include phosphate-based or silicon-containing compounds that promote robust CEI formation. A study by Cheng et al. highlights the dual role of tris(trimethyl-silyl) phosphite (TMSP) as an electrolyte additive in DIBs [138]. As detailed in Fig. 4f, the primary function of TMSP is to act as an oxidative sacrificial agent, undergoing preferential decomposition to engineer a robust and stable CEI layer rich in inorganic species like SiO^−^ and PO_2_^−^, which consistent of TOF-SIM and HRTEM results. This superior film effectively passivates the graphite cathode, preventing its direct contact with the electrolyte and the subsequent side reactions. A secondary, but still crucial, benefit is TMSP ability to inhibit the generation of harmful byproducts such as HF, which further contributes to CEI stability. The resulting thin, uniform, and stable CEI layer enables a remarkable lifespan of over 5000 cycles with a high capacity of 90.1 mAh g^−1^ at 30 C. Beyond TMSP, numerous additives are used in traditional LIBs to scavenge harmful byproducts like HF from LiPF_6_ decomposition. These additives, which contain Lewis base groups or reactive sites, neutralize acidic species and radical intermediates to mitigate cathode corrosion and preserve interfacial integrity [140]. In addition, the synergistic combination of multiple additives is also under investigation to achieve multi-functional protection across a wide voltage range. For instance, Ru et al. developed a dual-additive strategy using lithium difluoro bisoxalate phosphate (LiDFBOP), and 1,3-divinyl-1,1,3,3-tetramethyldisilazane (DTDS) to enhance high-voltage stability [141]. LiDFBOP regulates Li⁺ solvation to enable robust CEI formation, while DTDS scavenges impurities such as HF and H_2_O.

Moreover, to retard the premature electrolyte oxidation induced by the catalytic activity of graphite edges, surface coating and artificial CEI strategies are frequently implemented. For instance, coating graphite with a nanometric layer of amorphous implanting polyphosphoric acid (PPA) has been shown to effectively passivate these high-energy defects, physically decoupling the solvent from catalytic sites while maintaining anion transport channels [142]. Similarly, encapsulating graphite with amorphous carbon layers can not only shield the active edge planes but also buffer the volume expansion during anion intercalation, thereby significantly extending the cycling lifespan of DIBs [143]. Enhancing the oxidative stability of electrolytes is inherently a system-level endeavor, it is governed not only by the intrinsic properties of the electrolyte components but also by the interactions and coupling among the other cell components.

### Strategies for Suppressing Solvent Co-Intercalation

In strongly polar solvents, anions such as PF_6_^−^ or TFSI^−^ are tightly solvated, resulting in stable solvation shells that are difficult to strip during the intercalation process. This high desolvation energy barrier increases the likelihood that the entire solvated anion–solvent complex enters the cathode host, leading to co-intercalation. To address this issue, two complementary strategies have been developed: the design of weakly solvating solvents to reduce solvation shell stability, and the construction of dense, selective CEI to physically block co-intercalating species.

**Fig. 5:**
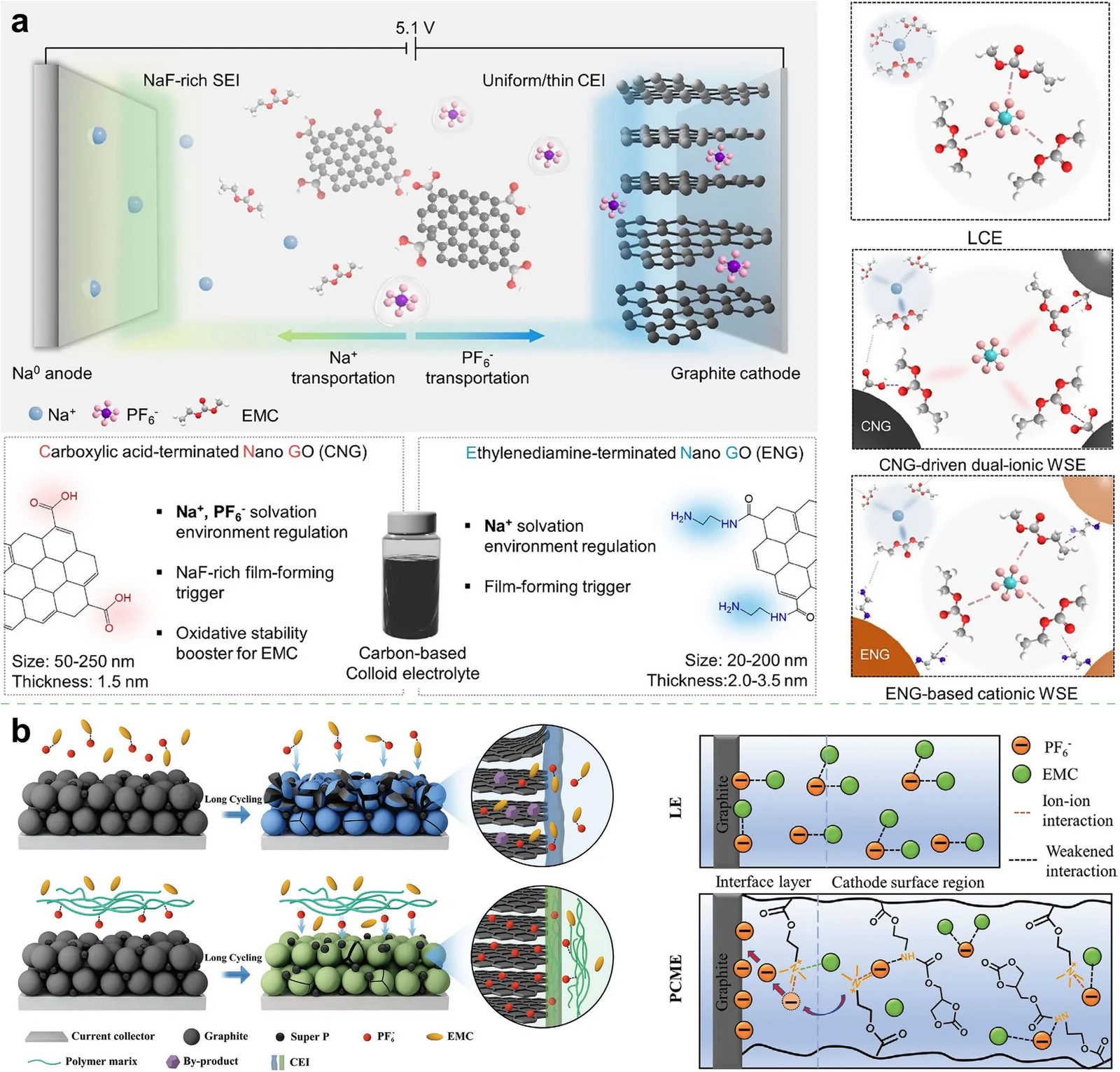
**a** Schematic of the electrolyte design strategy of dual-ionic weakly solvating electrolytes using carbonaceous colloids for anion shuttle batteries; CNG for DWSE; ENG for CWSE; Schematic of the solvation environments of BE, DWSE, and CWSE [147]. Copyright 2025, Wiley–VCH. **b** Schematic illustration of the EMC co-intercalation behavior comparison in LE or PCME based DIBs; Schematic illustration of LE and PCME solvation structure [148]. Copyright 2022, Wiley–VCH

The primary and most direct strategy to suppress solvent co-intercalation is to modulate the solvation behavior of anions by designing solvents with intrinsically weak coordinating ability. Distinct from the physical dilution of LHCEs that relies on inert diluents to maintain local anion-rich solvation structure, this approach represents an intrinsic solvent engineering. Researchers have focused on low-polarity, low-donor-number solvents, such as highly fluorinated ethers, esters, and nitriles, to spontaneously induce ion pairing and construct a similarly beneficial AGGs and CIPs structures without the need for auxiliary diluents. Most critically, in the context of DIBs, the concept of weak solvation is not merely restricted to cations. Weakly solvating solvents can significantly reduce anion–solvent interaction between anion and solvent, thereby lowering the desolvation energy barrier at the cathode interface [144–146]. This promotes the selective intercalation of bare anions while minimizing the co-transport of solvent molecules. Yao et al. first proposed the concept of a weakly solvating electrolyte (WSE), employing a pure non-polar solvent to create a solvation environment dominated by ion pairs and aggregates even at low salt concentrations [144]. This solvation structure promotes the formation of anion-derived interphases on graphite, enabling fast-charging capability and long-term cycling stability. Their used of Raman spectroscopy for the electrolyte bulk has also emerged as a powerful instance for probing and identifying solvation structures. Additionally, some design strategies incorporate polar or functional groups into the solvent structure. These groups enhance intermolecular interactions between solvent or additive molecules, which weakens the bind between the solvent and both cations and anions, creating a weakly solvating environment to suppress co-intercalation. For instance, Kang et al. developed a dual-ionic weakly solvating electrolyte by incorporating colloidal electrolytes prepared with nano-graphene oxides (NGOs) functionalized with distinct surface functionalities as shown in Fig. 5a [147]. The ethylenediamine-passivated NGOs (ENGs) were used to create a cationic weakly solvating electrolyte (CWSE), while carboxylic acid-functionalized NGOs (CNGs) established a dual-ionic weakly solvating electrolyte (DWSE). These functionalized NGOs disrupt the conventional solvation structures of Na^+^ and PF_6_^−^ ions by strongly interacting with the solvent, thereby promoting ion desolvation and transport while preventing solvent co-intercalation. As a result, the DWSE cell delivered a reversible capacity of 82.0 mAh g^−1^ at 50 C and stable cycling for over 1500 cycles at 10 C, showcasing excellent performance for Na-based DIBs. It is worth noting that while WSE hold the potential to circumvent the economic burden and environmental footprint of fluorinated diluents inherent to LHCE, they face a critical thermodynamic dilemma. The requisite weak coordinating ability inevitably compromises salt solubility and ionic conductivity. Consequently, unlike the physical blending in LHCE, WSE demand a significantly more sophisticated molecular design strategy to create solvents that are weak enough to liberate anions for interphase formation yet strong enough to prevent salt precipitation, representing a higher tier of electrolyte innovation.

A complementary and equally critical strategy lies in controlling the properties of the CEI. An appropriately engineered CEI functions analogously to a molecular filter, enabling anion conduction but impeding the passage of solvated clusters. To achieve this, the CEI must be mechanically robust, structurally compact, and ion selective. This often requires tuning the interfacial chemistry through solvent decomposition pathways or through the inclusion of functional additives that guide the formation of a desirable CEI composition [149, 150]. Specifically, inorganic-rich CEI layers containing fluorine, or oxygen-based species have shown promise in enabling anion conduction while blocking solvent infiltration [151, 152]. These can be generated through the decomposition of carefully selected solvents or salt components, or via sacrificial additives that decompose preferentially to form the desired interphase. For example, Liu et al. introduces a pre-constructed artificial SEI effectively preventing the co-intercalation of anions and enhancing cycling stability [153]. The artificial SEI mainly included NaF, Na_2_CO_3_, ROCO_2_Na derived from the decomposition of carbonate electrolyte and sodium salt, suppresses irreversible phase transitions and graphite exfoliation, while significantly broadening the voltage window of carbonate electrolytes, leading to improved capacity and excellent retention over 100 cycles at 4.6 V high operating voltages. In some cases, polymer components in the electrolyte can form crosslinked organic–inorganic hybrid films that enhance the CEI’s selectivity and structural integrity. The functional groups on the polymer backbone play a crucial role in enhancing selectivity by selectively interacting with specific ionic or solvent species, thereby modulating the ion solvation structures. As shown in Fig. 5b, a robust CEI was developed by an anion-permselective polymer electrolyte with cationic quaternary ammonium groups, named PCME [148]. By strong interaction with solvent, the polymer functional groups facilitate PF_6_^−^ desolvation, significantly inhibiting solvent co-intercalation and improving the electrolyte’s oxidation resistance. Compared with normal system which name LE, the well-constructed CEI by PCME ensures the structural integrity of graphite and enhances cycling stability, with the graphite||Li cell achieving 87.1% capacity retention after 2000 cycles at a high cutoff potential of 5.4 V and a Coulombic efficiency of 99.0%.

### Strategies for Stabilizing Electrode–Electrolyte Interphase

In DIBs, particularly when graphite is employed as the cathode material, the stability of the electrode–electrolyte interphase is critical for ensuring long-term performance and safety [154]. This is largely attributed to the high operating voltages and the transport of bulky anions, which induce severe lattice expansion and mechanical stress on the cathode. Interfacial stabilization strategies generally fall into two main categories including in situ formation of stable interphase layers via electrolyte engineering and *ex-situ* surface modifications, such as artificial solid-electrolyte interphases or protective cathode coatings.

**Fig. 6:**
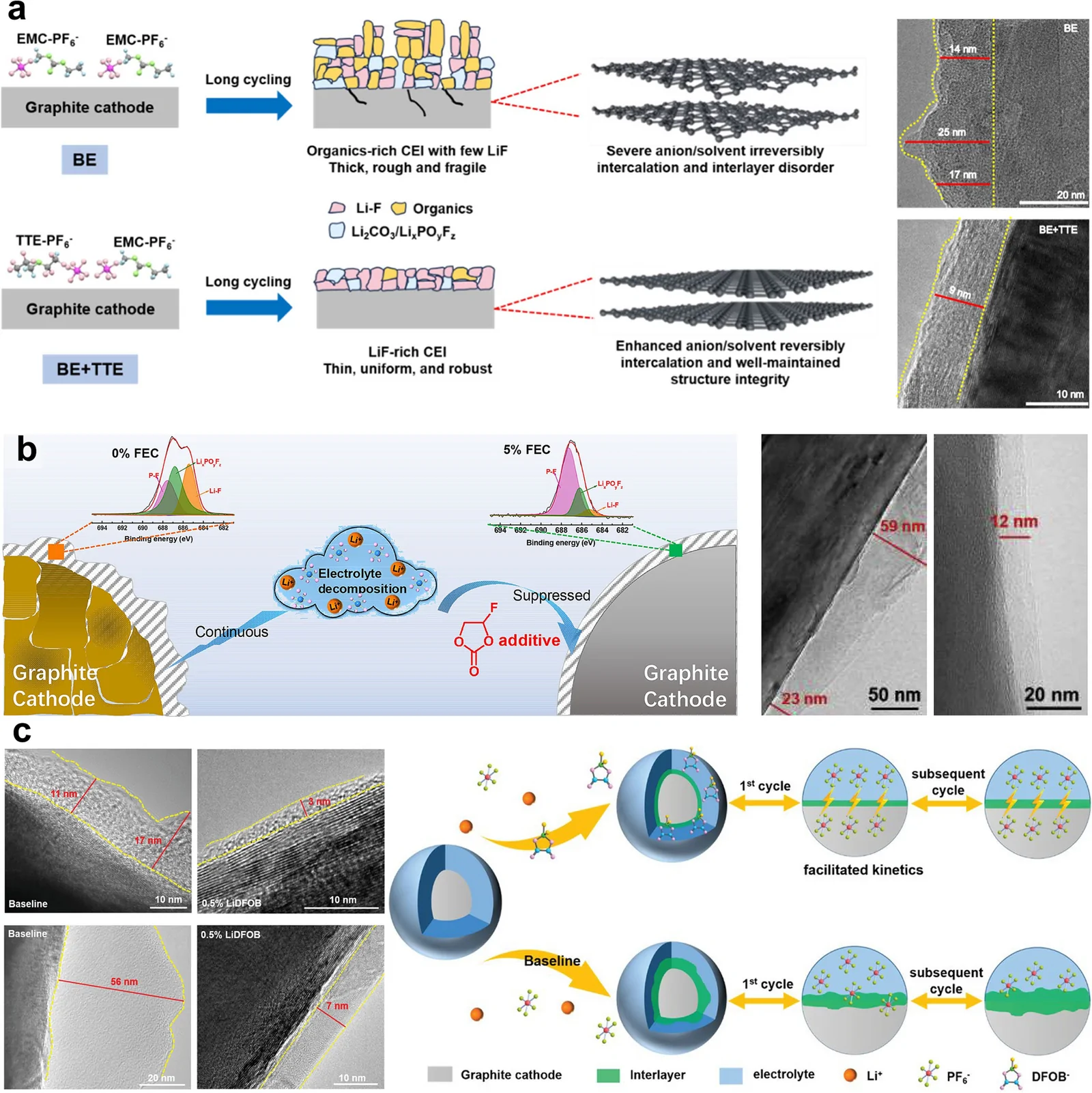
**a** Schematic illustration of the problems induced by the basic electrolyte and the stable mechanism with TTE additive**;** HRTEM images of the graphite surface after 100 cycles with BE and BE + TTE [155]. Copyright 2024, Elsevier. **b** Schematic illustration of FEC additive and XPS spectrum. TEM images of graphite electrodes at the fully charged state after 200 cycles in electrolyte with 0% FEC and 5% FEC [156]. Copyright 2020, Elsevier. **c** TEM images after the 2nd and the 150th cycle using baseline electrolyte and 0.5 wt% LiDFOB-containing electrolyte; Illustration of the CEI evolution during cycling in electrolyte with and without LiDFOB additive [157]. Copyright 2023, Wiley–VCH

Electrolyte engineering centers on the precise tuning of electrolyte components to enable the spontaneous formation of robust and ionically conductive interphases at the electrode surfaces during battery operation [48]. For the DIBs, particularly under high-voltage conditions, graphite cathodes tend to facilitate the oxidative decomposition of electrolytes, resulting in unstable CEI layers. Given the typically large size of anions involved in DIBs, the CEI must not only be permeable to bulky anions but also effectively suppress further electrolyte degradation. This can be achieved through the incorporation of functional components, enabling their preferential decomposition or polymerization at the cathode surface to form stable inorganic or hybrid organic–inorganic interphases enriched with elements such as fluorine or phosphorus. Wu et al. introduced TTE as an anion-focused electrolyte additive for DIBs, demonstrating its ability to enter the PF_6_^−^ solvation shell in basic electrolyte, thereby facilitating the formation of stable interphase [155]. By promoting the formation of a LiF-rich CEI on the graphite cathode, TTE effectively suppresses electrolyte decomposition and structural degradation during long-term cycling, as shown in Fig. 6a. Furthermore, the stability of this TTE-dominated CEI is visually confirmed by the HRTEM image for cathode surface after 100 cycles. As a result, the Li||graphite cell delivers excellent cycling stability with 67.6% capacity retention over 5000 cycles and a high-rate capability of 95.6% at 30 C. In addition, by introducing FEC as an electrolyte additive in graphite-based DIBs, a uniform and LiF-deficient CEI is formed on the graphite surface shown in Fig. 6b [156]. This CEI effectively suppresses electrolyte decomposition and structural degradation of the cathode, thereby enabling highly reversible PF_6_^−^ (de)intercalation. As an established film-forming additive in conventional LIBs, lithium difluoro(oxalate) borate (LiDFOB) as a functional additive introduced to tailor the interfacial chemistry of graphite-based DIBs [157]. With only 0.5 wt% addition, LiDFOB promotes the in-situ formation of a highly conductive and protective CEI displayed in Fig. 6c, which enhances PF_6_^−^ intercalation kinetics, mitigates parasitic reactions, and reinforces structural stability of the cathode by forming a robust cathode-electrolyte interface capable of accommodating lattice breathing. As a result, the modified system achieves outstanding fast-charging capability and long-term cycling, maintaining 87.5% capacity over 4000 cycles and delivering energy and power densities up to 422.7 Wh kg^−1^ and 18.8 kW kg^−1^, respectively. Moreover, the selection and concentration of solvents and salts can significantly influence CEI formation. Zhao et al. explores the impact of LiFSI-based electrolytes in FEC/FEMC solvents at varying concentrations (1, 3, 5, and 6 mol L^−1^) on graphite-based DIBs performance, emphasizing the role of the CEI [158]. While higher salt concentrations improve electrolyte stability and suppress corrosion, they also lead to thicker and less effective CEI layers, increasing polarization and reducing rate performance. The optimal 3 mol L^−1^ LiFSI concentration effectively balances CEI formation, ionic transport, and electrode stability, resulting in the best electrochemical performance.

**Fig. 7:**
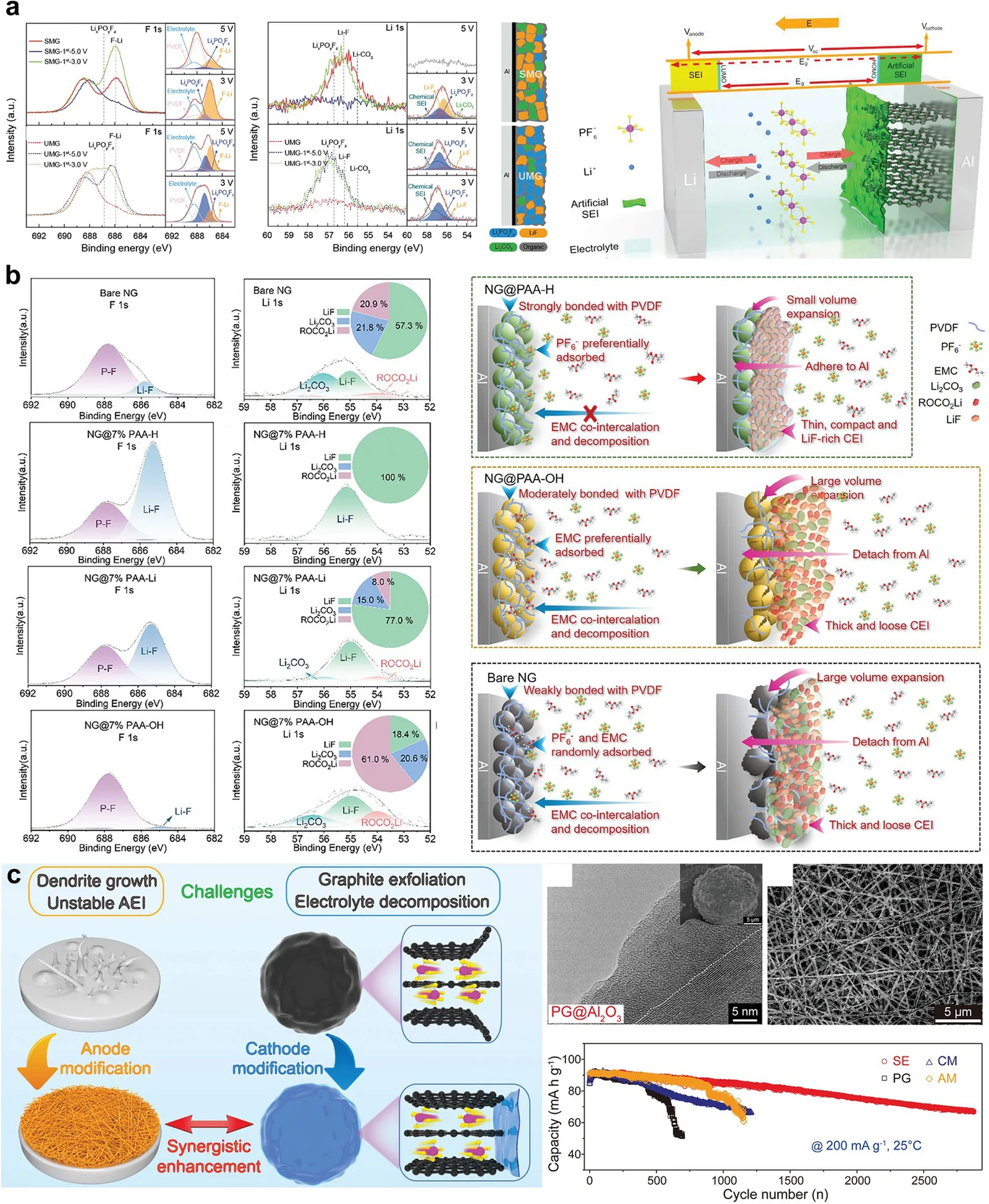
**a** XPS spectra of F 1*s*, Li 1*s* of SMG and UMG during the first cycle at different states, and the corresponding deconvolution of the charged state of 5.0 V and the discharged state of 3.0 V; The scheme illustration displays the working mechanism of DIB with artificial CEI [160]. Copyright 2019, Wiley–VCH. **b** Deconvoluted XPS spectra of NG, PAA-H, PAA-Li, and PAA-OH; The schematic illustration of the functions of PAA modifier on NG [162]. Copyright 2025, Wiley–VCH. **c** Schematic illustration of the main challenges in LG-DIBs, and the proposed two-pronged strategy to address them; TEM image of PG@Al_2_O_3_; SEM image of the ESF; Typical cycling performance of PG, AM, CM, and SE [159]. Copyright 2022, Wiley–VCH

The artificial SEI/CEI strategy involves the deliberate deposition of protective layers on the electrode surface prior to cell assembly. This *ex-situ* approach avoids the complexities associated with *in-situ* interphase formation and offers the potential for more precise control over interfacial properties [159]. For the cathode, one strategy involves constructing artificial CEI layers in advance through chemical or electrochemical treatments, enabling protective films to form directly on the electrode surface. For instance, Wu et al. constructed an artificial CEI layer deliberately formed on the graphite cathode by applying a controlled electrochemical pretreatment [160]. Further analysis via XPS spectra in Fig. 7a reveals that this pretreatment SEI-modified graphite (SMG) forms a protective CEI that significantly suppresses electrolyte decomposition compared with the unmodified graphite (UMG), thereby enhancing the reversible capacity and stabilizing the electrode. As a result, the modified graphite displayed, delivers a higher specific capacity of ~ 84.5 mAh g^−1^ at 200 mA g^−1^ and exhibits markedly improved cycling stability compared to the untreated electrode, even up to a cutoff voltage of 5.0 V. This approach often results in good adhesion and mechanical robustness, offering reliable protection during cycling.

Another common method is to apply a thin layer of inert materials, such as metal oxides or polymer, onto the electrode surface. For example, to mitigate high-voltage-induced cathode degradation in cathode, a robust Al_2_O_3_ interphase was constructed on graphite to alleviate structural damage caused by repeated anion extraction [160]. The electrically insulating nature of Al_2_O_3_ further suppresses parasitic reactions by impeding the accumulation of decomposition products. Moreover, Wu et al. also engineered a uniform artificial CEI constructed on the graphite cathode through a wet-chemical Al_2_O_3_ coating method using Al_2_(SO_4_)_3_ in a buffered aqueous solution [161]. This conformal oxide layer effectively curbs electrolyte decomposition and promotes the formation of a more elastic CEI with reduced inorganic content. Consequently, the treated electrode retains 82.3% of its capacity after 1000 cycles. Polyacrylic acid (PAA), employed as a surface modifier by Wang et al., plays a multifunctional role by utilizing its polymer elasticity to buffer the massive volume variations of graphite, reinforcing the adhesion between natural graphite (NG) and PVDF binder, and simultaneously regulating interfacial chemistry [162]. Its acidic functional groups facilitate the formation of a compact and LiF-enriched CEI layer. XPS analysis in Fig. 7b provides strong evidence for this mechanism, revealing significant differences in CEI composition among cathodes which include that the PAA coated NG materials obtained under acidic, neutral and alkaline conditions were denoted as NG@PAA-H, NG@PAA-Li and NG@PAA-OH, respectively. The NG@7%PAA-H cathode exhibited the highest relative intensity of the LiF peak, accounting for nearly 100% of the Li-related CEI components. This high LiF content suggests that the acidic PAA-H coating preferentially adsorbs and facilitates the decomposition of PF_6_^−^ anions. In stark contrast, cathodes such as the untreated NG and NG@7%PAA-OH showed a remarkable increase in solvent decomposition products like Li_2_CO_3_ and ROCO_2_Li. This indicates that without the acidic PAA-H coating, solvent co-intercalation and decomposition are severe. The optimized NG@7%PAA-H cathode, therefore, delivered excellent durability with 73.9% capacity retention over 8000 cycles, with the LiF-rich CEI effectively suppressing EMC decomposition.

For artificial SEIs on the anode side, including inorganic coatings such as Al_2_O_3_, LiPON or LiF [163], and polymer-based coatings such as those derived from polyethylene oxide (PEO) [164] or polyvinylidene fluoride (PVDF) [165] can be applied, these interfacial engineering strategies also converge with those widely adopted in conventional LIBs. Artificial layers are typically stable against electrolyte reduction, serve as effective barriers to electron transport while remaining permeable to ions, thereby mitigating undesirable side reactions and enhancing cycling stability. To address the issue of dendrite growth, an artificial SEI via a nitrogen-doped electrospun film (ESF) was constructed on the lithium metal anode as shown in Fig. 7c, which not only regulates uniform Li deposition and buffers volume fluctuation, but also synergistically suppresses electrolyte decomposition and graphite exfoliation, enabling stable DIBs operation [159]. To determine the working mechanisms of the two-pronged strategy, four DIBs were assembled using pristine graphite (PG), cathode modification (CM), anode modification (AM), and synergistic enhancement (SE). While both CM and AM individually improved capacity retention, the SE approach, which combines both modifications, achieved a remarkable cycle life of 2700 cycles with 80% capacity retention. Analysis revealed that AM is critical for maintaining a high and consistent CE, whereas CM primarily suppresses high-voltage electrolyte decomposition. The dual optimization strategy can significantly improve the cyclic stability of LG-DIBs.

### Strategies for Balancing Electrode Kinetics

High-rate capability is one of the most promising advantages of carbon-based DIBs. To fully realize this potential, balancing the kinetics of both the cathode and the anode is an indispensable prerequisite. Four critical kinetic processes remain to be addressed aimed to overcome the kinetic bottleneck on the anode side, which include desolvation of solvated metal ions, charge transfer at the electrode–electrolyte interphase, transport of alkali metal ions across the SEI layer, and solid-state diffusion in the anode [37]. Shifting the research focus from anions to alkali metal cations, the design principles for weakly solvating electrolytes, commonly used in LIBs, are considered highly relevant and transferable to DIBs, providing solutions to address the kinetic bottleneck on the anode side from the perspective of reducing the desolvation barrier of cations.

**Fig. 8:**
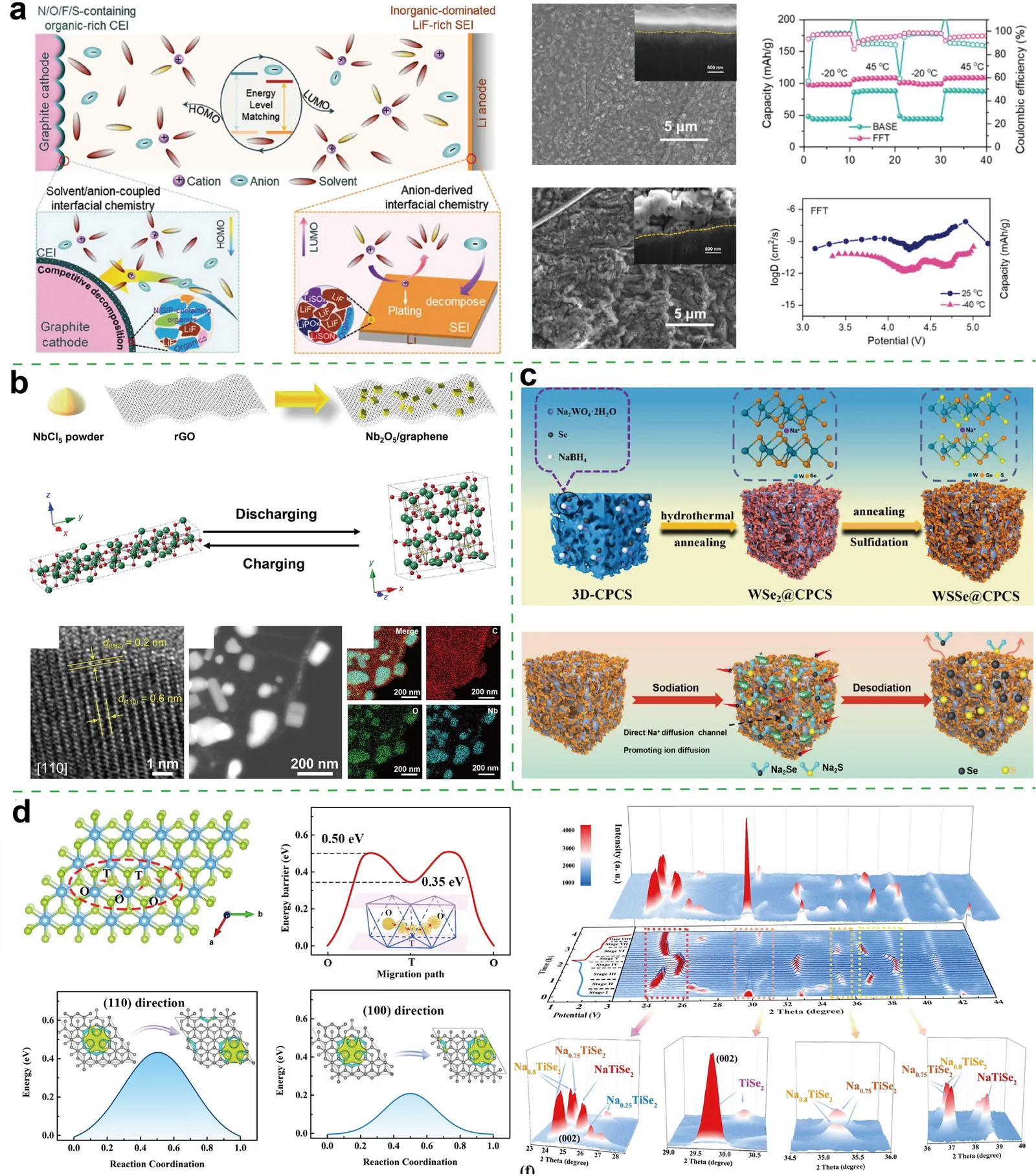
**a** Scheme illustration for energy level adapting enabled solvent/anion-coupled interfacial chemistry on the cathode and anion-derived interfacial chemistry on the anode; Morphological SEM images of Li metal anode after 100 cycles using FFT and BASE electrolytes; Specific capacities of the FFT and BASE-type DIBs alternatively operated at -20 °C (1 C) and 45 °C (5 C); Ion-diffusion coefficient derived from GITT discharge measurements of the FFT-type Li||graphite DIB at room temperature and -40 °C [166]. Copyright 2024, Wiley–VCH. **b** Scheme illustration of the synthesis procedures of Nb_2_O_5_/G; Schematic illustration of Nb_2_O_5_/G lithium storage mechanism; The inverse fast Fourier trans (IFFT) image; HAADF-STEM image; Corresponding EDS elemental mapping of Nb_2_O_5_/G 167]. Copyright 2024, Springer. **c** Illustration of the formation process of WSSe@CPCS; The schematic graph of WSSe@CPCS electrode composite upon cycling [168]. Copyright 2023, Wiley–VCH. **d** Proposed Na-ion diffusion path, diffusion channel and energy barriers in TiSe_2_, respectively; The diffusion energy barrier of PF_6_^−^ anions along the (100) and (110) directions of graphite; Unraveling the reaction mechanism during sodiation-desodiation process of TiSe_2_ electrodes; In situ XRD survey with a close up on the 22.0°–44.0° diffraction range [169]. Copyright 2021, Wiley–VCH

Compared to selectivity and high-voltage stability, the SEI design here focuses more on high ionic conductivity and uniformity, ensuring that ion transport across the interphase does not become a kinetic bottleneck. Similarly, the SEI strategies adapted for high-rate LIBs can serve as valuable references for DIBs. Lang et al. emphasized the design of anion-derived SEI on lithium metal via an energy-level-adaptive electrolyte design in Fig. 8a, where a uniform and smooth surface in contrast to rough surface formed with base electrolyte (BASE), was constructed to ensure fast and stable Li⁺ plating/stripping even at − 40 °C [166]. The electrolyte, designated as FTT, was rationally formulated by dissolving 1 mol L^−1^ LiFSI in a solvent mixture of FEMC and non-flammable tri(2,2,2-trifluoroethyl) phosphate (TFEP). The SEI formed by FFT exhibits interfacial kinetics that match well with the engineered organic-rich CEI on the graphite cathode even in low-temperature, together enabling DIBs to operate efficiently across a wide temperature range with high power capability.

In addition to the intensively explored electrolyte and interphase engineering, the solid-state diffusion of ions in electrode materials, particularly within the anode, has emerged as a key limiting factor in resolving the kinetic mismatch between the cathode and anode. Taking graphite-based DIBs as an example, PF_6_^−^ diffusion in graphite cathode exhibits a relatively low energy barrier of ~ 0.2 eV, while Li^+^ and K^+^ diffusions face significantly higher barriers of 0.42–0.52 eV [82] and 0.11–1.58 eV [83], respectively, causing kinetic asymmetry between the electrodes that hampers high-rate capability and overall cell stability. Such disparities in ionic mobility highlight the imperative of rational anode material engineering, including strategies such as heteroatom doping and interlayer spacing modulation, to enhance ionic conductivity and achieve balanced ion transport kinetics across both electrodes. Figure 8b displays that Lei et al. focused on enhancing graphite performance by coupling it with Nb_2_O_5_ nanocrystals/graphene composites (Nb_2_O_5_/G), which significantly improve ionic transport [167]. HRTEM, IFFT patterns, STEM and elemental mapping images indicated the successful coupling and assembly of Nb_2_O_5_ nanocrystals and graphene at the atomic scale. The high conductivity of graphene and the nanoscale structure of Nb_2_O_5_ facilitated efficient charge and discharge processes, leading to enhanced electrochemical performance via unique lithium storage mechanism. In addition, a WSSe nanosheets on a 3D porous carbon skeleton was fabricated as anode shown in Fig. 8c, which enhances Na⁺ adsorption and significantly boosts electronic conductivity through S-doping. The interconnected porous structure of CPCS helps balance the anode’s ion diffusion kinetics with the cathode, leading to improved cycling stability and rate capability in sodium-ion batteries by optimizing the overall electrochemical dynamics [168].

Moreover, a TiSe_2_-graphite Na-DIBs was introduced featuring a high-rate anode material with expanded interlayer spacing and metallic conductivity [169]. In situ XRD reveals a multi-phase and reversible sodiation mechanism which involved sequential transitions through Na_0.25_TiSe_2_, Na_0.75_TiSe_2_, Na_0.8_TiSe_2_, and NaTiSe_2_, thereby confirming the structural adaptability of TiSe_2_ during Na^+^ intercalation as shown in Fig. 8d. Benefiting from its low Na^+^ diffusion barrier and fast diffusion coefficient, the TiSe_2_ anode supports rapid Na^+^ transport kinetics that are comparable to the anion diffusion in graphite cathodes, thereby mitigating kinetic asymmetry and facilitating balanced ion transport across electrodes. MXenes, a family of 2D transition metal carbides/nitrides, feature conductive layered structures and tunable surface chemistry, offering high potential as fast-charging anodes that can be coupled with anion-intercalation graphite. Sabaghi et al. developed a high-rate K^+^-intercalation anode by grafting multifunctional azobenzene sulfonic acid onto V_2_C MXene, forming ASA-V_2_C [170]. This molecular modification strategy not only increased the interlayer spacing from 0.86 to 1.25 nm but also introduced additional K^+^-hosting sites and fast ionic transport pathways. The resulting electrode exhibited markedly improved specific capacity and high-rate performance compared to unmodified V_2_C. Notably, the soft organic grafts served as mechanical buffers, mitigating structural stress during cycling and enhancing long-term stability displayed 80.3% capacity retention after 900 cycles.

### Strategies for Pursuing High Capacity and High Energy Density

**Fig. 9:**
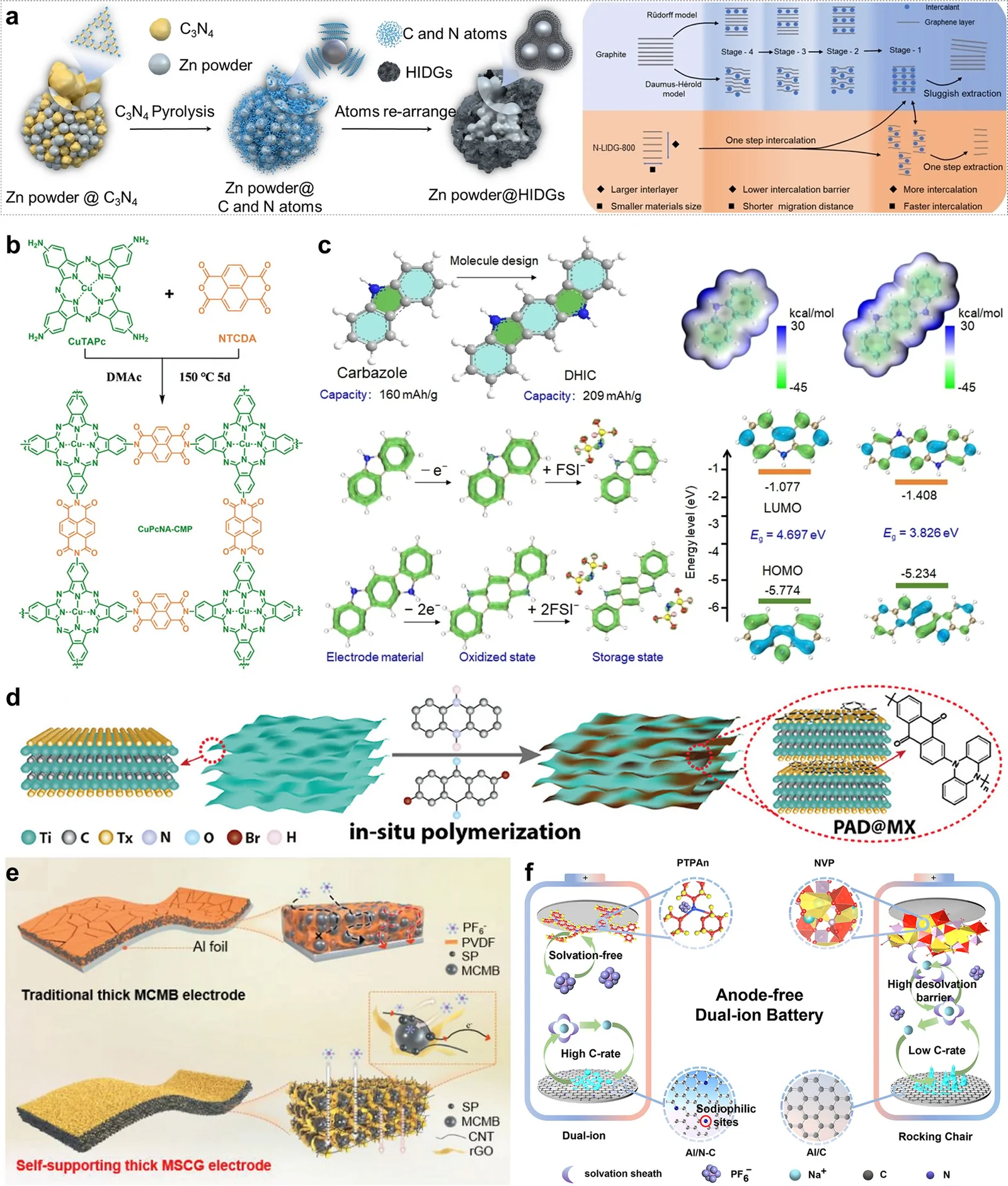
**a** Illustration of synthesis of N-LIDGs and the illustration of the different work mechanisms of graphite and N-LIDG-800 in DIBs [173]. Copyright 2023, Wiley–VCH. **b** Synthesis and structure of CuPcNA-CMP [184]. Copyright 2021, Wiley–VCH. **c** Molecular design conception from carbazole to DHIC, MESP-mapped molecular van der Waals surface of carbazole and DHIC, LOL-π mappings of carbazole and DHIC after the oxidized and anion storage states, and energy diagrams of carbazole and DHIC [186]. Copyright 2024, Wiley–VCH. **d** Synthesis of PAD@MX [115]. Copyright 2023, Wiley–VCH. **e** Schematic illustration of the structures of the MCMB and MSCG electrodes and the corresponding transportation of electrons/PF_6_^−^ ions in the high-mass-loading electrodes during cycling [88]. Copyright 2023, Wiley–VCH. **f** Schematic illustration of AFSDIB (left) and traditional “rocking chair” anode-free sodium metal battery (right). The application of solvation-free PTPAn cathode and sodiophilic Al/N–C collector in the AFSDIB is favorable for achieving high C-rate [210]. Copyright 2024, Elsevier

Traditional graphite has shown the ability of large-sized anion storage in different electrolytes despite its relatively low theoretical specific capacity. To pursue higher reversible capacity and energy density, the structural regulation and morphological modification of the graphite is imperative to boost the practical capacity [171]. Compared to fully graphitized carbon, locally ordered graphite carbons (LOGC) have reduced layer size, which can weaken the van der Waals interaction between adjacent graphene sheets, meanwhile, the disordered regions not only provide numerous capacitive anion storage sites but also interconnect the dispersed nanographite, improving the structural stability [172]. Commercial ketjen black (KB) is a typical LOGC, which has both a graphite region with an enlarged interlayer spacing of about 0.356 nm and an amorphous region that offers a large specific surface area for extra anion adsorption. When the KB was used as cathode for potassium-based DIB with electrolyte of 1 M KPF_6_ in EC/PC (1:1, v: v), the cathode delivered an ultrahigh specific capacity of 232 mAh g^−1^ at 50 mA g^−1^ in a voltage window from 1.5 to 4.6 V, indicating the effectiveness of this structural design strategy in enhancing capacity, although the operating voltage inevitably decreased. As shown in Fig. 9a, N-doping graphite with ultra-large interlayer distance (N-LIDGs) can be prepared by a one-step pyrolysis process of g-CN under the catalysis effect of metallic Zn powder [173]. The N-LIDG treated at a temperature of 800 °C exhibited a thin-layered graphene morphology with a large interlayer distance of 0.51 nm, which provided an enhanced adaptability and larger space for fast migration and storage of large-sized anions. In addition, the homogeneous doping of N element in carbon nanosheets effectively reduced the energy barriers for anion intercalation promoting the ionic adsorption. Different conventional Rudörff or Daumus-Hérold models, the expanded interlayer and abundant storage sites enable N-LIDG-800 to accommodate more anions via a special “deep-breathing” mechanism, consequently showing a high capacity of 240 mAh g^−1^.

Besides graphite-based cathodes, researchers have also explored alternative cathode materials with high theoretical specific capacity for anion storage [174–178]. With advantages in structural designability, environmentally benign, and abundant resources features, organic materials are emerging as promising candidates for next-generation secondary batteries [179, 180]. By designing electroactive redox functional groups, various types of organic electrode materials such as *n*-, *p*-, and bipolar-type ones have been developed [181]. Particularly, bipolar-type organic materials integrate the properties of both *n*-, and *p*-type materials, which can accommodate cations and anions during discharge and charge process, respectively. For example, phthalocyanine and its derivatives (Pcs) are typical bipolar-type organics, which can be reduced to accept electrons (20 π-electrons) or oxidized to donate electrons (16 *π*-electrons) due to their large delocalized π conjugated macrocyclic [182, 183]. As shown in Fig. 9b, Wang et al. reported the synthesis of Pc-based CMP (named as CuPcNA-CMP) via the imidization reaction of copper (II) tetraaminephthalocyanine (CuTAPc) and 1,4,5,8-naphthalenetetracarboxylic dianhydride (NTCDA) [184]. In this design, both CuTAPc and NTCDA could serve as not only redox-active centers to contribute to the capacity, but also the building blocks and linker groups to link each other. The CuPcNA-CMP sample delivered a high reversible capacity of 202.4 mAh g^−1^ at 0.2 A g^−1^ at a voltage window of 1.5–4.5 V, which may result from the rapid multiple redox reactions. Although the cathode showed a high initial capacity, it suffers from fast capacity decay with a capacity retention of 50% after 110 cycles. PQPZ is another bipolar polymer, which is composed of the electron-donating dihydrophenazine and the electron-withdrawing 9,10-phenanthraquinone [185]. The phenazine unit in PQPZ is p-type that can store anions through a two-electron redox reaction, while the 9,10-phenanthraquinone can store sodium. As the PQPZ is employed as cathode, four reversible redox peaks can be observed in CV curves in a voltage range of 1.0–4.0 V, in which the redox peaks located at 1.6/2.1 and 2.4/2.6 V belong to Na^+^ (de)intercalation processes, while redox peaks at 2.93/3.06 and 3.72/3.82 V correspond to the PF_6_^−^ (de)intercalation processes. As a result, the PQPZ can achieve a high specific capacity of 270 mAh g^−1^ and high energy density of 696 Wh kg^−1^ in a half-cell, emphasizing the feasibility of achieving high capacity and high energy density in the anion involved DIBs. Although organic materials exhibit high specific capacity in anion storage, small organic molecules suffer from low electronic conductivity, and unsatisfactory structure durability in organic electrolytes, hampering the battery cycling performance. Synergetic molecular engineering strategy involves the dual extension of π-conjugated motifs with active sites, which may effectively harness the electronic delocalization effect of electron-withdrawing conjugated groups to enhance electronic conductivity, and enhance the electrochemical stability. For example, 5,11-dihydridoindolo[3,2-b]carbazole (DHIC) provides abundant redox-active centers and polymerization anchors with its elongated carbazole groups [186]. The dual elongated π-conjugated system, incorporating both the carbazole motif and electropolymerized DHIC (pDHIC), offers an increased specific capacity, and enhanced anion storage stability with its robust nanonetwork polymer structures (Fig. 9c). As a result, the pDHIC cathode delivered a high capacity of 197 mAh g^−1^ at 50 mA g^−1^, and a high retention of 86.1% over 500 cycles at 300 mA g^−1^.

As an emerging 2D material, MXene has gained great attention due to its advantages such as abundant surface functional groups, high electrical conductivity, good hydrophilicity, high thermal stability, and excellent mechanical properties [187]. Benefiting from its unique structure and physicochemical properties, recently the applications of MXene in electrodes, current collectors, and separators across different types of batteries have significantly increased [188–197]. When the Ti_3_C_2_T_x_ MXene was employed as electrode material for DIBs with a wide voltage window of 1.0–5.0 V, the cathode displayed synergistic intercalation of both PF_6_^−^ and Li^+^ at different voltage ranges [198]. On the one hand, Li^+^ intercalation at low discharge voltage can expand the interlayer spacing of Ti_3_C_2_T_x_, meanwhile, the remaining Li^+^ during charging provides active sites to anchor PF_6_^−^ at high charging voltage. On the other hand, the residual PF_6_^−^ during discharge can act as active sites to coordinate with Li^+^, increasing the reversible capacity. Consequently, the Ti_3_C_2_T_x_ MXene delivered a high discharge capacity of 310 mAh g^−1^ at 200 mA g^−1^, which can be attributed to its electrochemical aging strengthening behavior. The combination of organic materials and MXene based on their advantages may provide an approach toward achieving high capacity and energy density. Furthermore, as shown in Fig. 9d, an organic bipolar polymer called poly[anthraquinone-alt-dihydrophenazine]@Ti_3_C_2_T_X_ MXene (PAD@MX) can be synthesized through a simple one-step polymerization method between the two-electron n-type unit of dibromoanthraquinone and two-electron p-type unit of dihydrophenazine on the layered MXene nanosheets [115]. The PAD@MX formed a unique layered structure with a large surface area, which not only offers numerous active sites but also effectively alleviates the volume expansion/contraction induced by the ion (de)intercalation, leading to its bipolar electrode performance in both Na-based and K-based DIBs. PAD@MX cathode delivered high discharge capacity of 240/255 mAh g^−1^ and energy densities of 511/597 Wh kg^−1^ in Na-based and K-based DIBs, respectively.

Although the reversible capacity of the cathode can be effectively improved via reasonable structural regulation strategies and new material discovery, excessive electrolyte is usually added along with ultra-thick separators and low mass loading of active material in half cell testing, resulting in a deep gap between academia and commercial production. Thus, it is urgent to increase the energy density of the DIBs through different strategies such as electrode structure design, high mass loading, special separator fabricating, and electrolyte design [199–202]. Conventional electrodes fabricated by a slurry casting onto metal foil, which not only have difficulties maintaining structural integrity during repeated charge/discharge processes due to the weak adhesion between the current collectors and the active materials but also suffer from high cost with metal current collectors. Wei et al. identified that an integrated free-standing functional all-carbon cathode (MSCG) can be rationally constructed by combining conductive 0D super-P nanoparticles, 1D carbon nanotubes, 2D reduced graphene oxide sheets, and 3D mesocarbon microbeads through a chemical coupling strategy without adding binders and current collectors (Fig. 9e) [88]. The MSCG showed “point-line-plane” hierarchical porous and conductive structure features, which provided well-interconnected ion/electron transport channels, promoting their diffusion kinetics and maintaining excellent mechanical properties. The MSCG cathode achieved the practical level of ultrahigh mass-loading (> 50 mg cm^−2^) and showed a high energy density of 379 Wh kg^−1^, promoting the development and practical applications of high-energy–density DIBs. Besides the electrode structural design, reducing the amount of electrolyte used is also crucial, since excessive solvent will induce side reactions and contribute parasitic weight. Typically, solvent-free electrolytes have revealed their effect in improving energy density. For example, by utilizing binary alkali metal molten salt based on bis(fluorosulfonyl)amide ([Na_0.56_K_0.44_][FSA]) as the electrolyte can avoid the usage of solvent, which effectively solved the issues induced by solvent molecules, such as irreversible decomposition, low Coulombic efficiency, co-intercalation, and reduced energy density [203]. The molten salt electrolyte can be fully utilized as an ion reservoir, in which Na^+^ and K^+^ work as cationic charge carriers while the FSA anion group was selected due to its ability to form a stable electrode–electrolyte interphase and the low melting points of its alkali metal salts. As a result, the NaK-DIB cell achieved stable operation in a temperature range of 90–120 °C with high theoretical energy densities of 246 Wh kg^−1^ and 533 Wh L^−1^ based on active materials and capacity-matched electrolyte. Anode-free strategy has witnessed its success in Li-based and Na-based batteries in improving the energy density [204–209]. A key challenge of this strategy is to suppress side reactions and to achieve highly reversible plating/stripping of Li or Na metals at the surface of the current collector. For instance, as shown in Fig. 9f, plasma-treated carbon-coated Al current collector (Al/N–C) performs a sodiophilic N-doped carbon surface, which effectively promotes the heterogeneous nucleation and deposition of Na and reversible Na stripping [210]. When Al/N–C was assembled with a PTPAn cathode, the anode-free Na-based DIBs displayed a remarkable energy density of over 380 Wh kg^−1^ and power density above 1800 W kg^−1^ based on active materials and consumed electrolyte, providing a new approach for developing high-energy DIBs.

### Strategies for Improving Low-Temperature Performance

The performance degradation at low temperatures can be attributed to the following factors, including the increased impedance of both cations and anions diffusion in bulk electrolyte, increased desolvation resistance at the surface of SEI or CEI, and sluggish kinetics of charge carriers’ migration through these electrode–electrolyte interphase [115, 211]. Electrolyte engineering is promising for enhancing the electrochemical performance at low temperatures [211, 212]. Fluorination to ester molecules effectively regulates the solvation structure, for example, difluoroester (-CHF_2_) not only enhanced the antioxidative property but also attenuated the anion-solvent interactions, which significantly reduced the anion desolvation kinetic barrier [22]. Thereby, the difluoro 2,2-difluroethyl acetate (DFEA)-based electrolyte demonstrated highly reversible and kinetically fast anion intercalation with high ionic conductivity (0.1–10.9 mS cm^−1^) across a wide range of temperatures (− 60 to + 60 °C). The anion intercalation lithium metal batteries based on this electrolyte yielded an excellent cycling stability over 3000 cycles without capacity degradation at − 20 °C, indicating the fast reaction kinetics of both PF_6_^−^ and Li^+^ at low temperature.

**Fig. 10:**
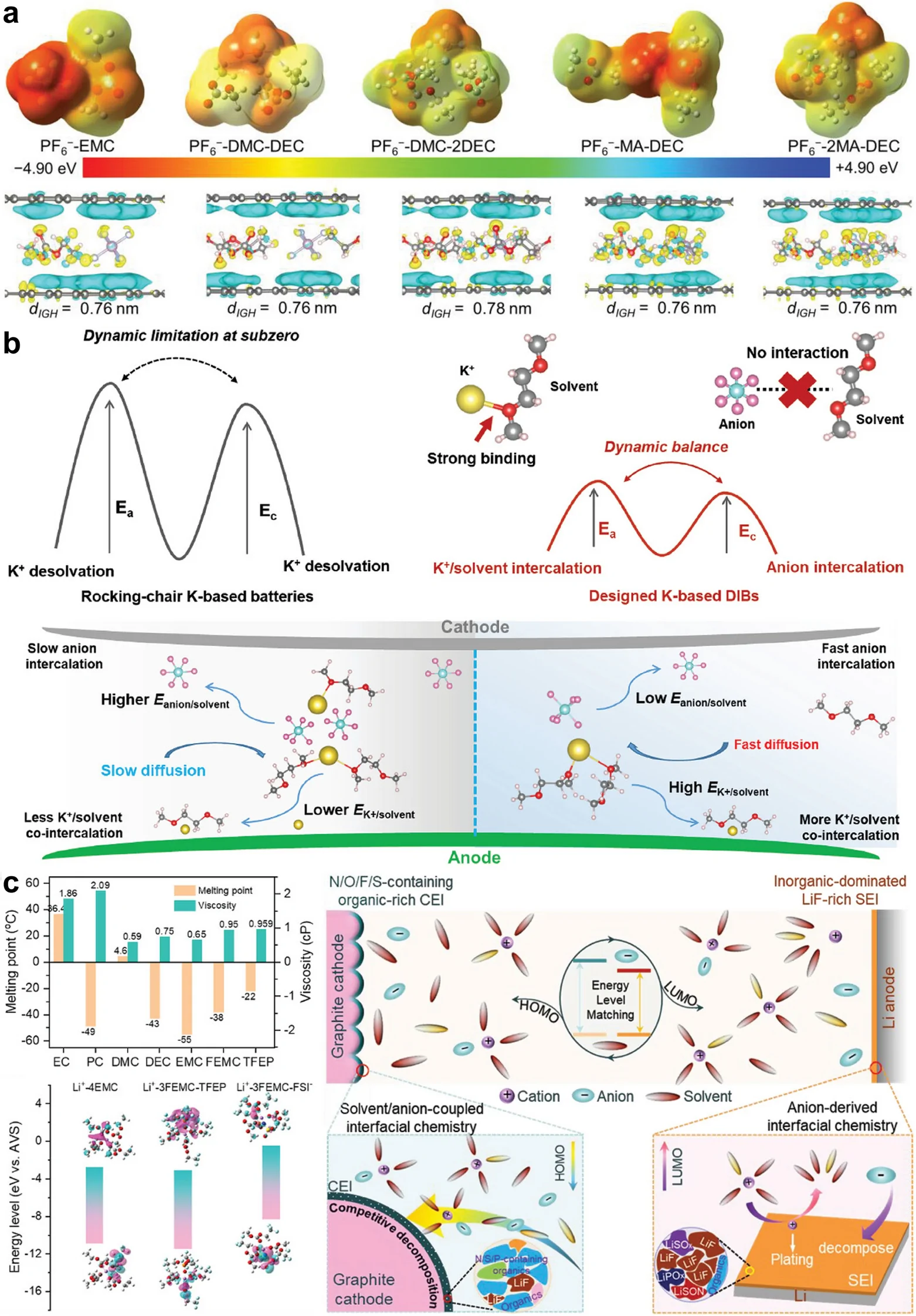
**a** Electrostatic potential mapping on electron distribution for the considered PF_6_^−^-solvent complexes. The charge density difference of AGICs with different intercalating species and the corresponding intercalated gallery height numbers (*d*_IGH_) gained from the optimized geometric structures. From left to right are PF_6_^−^-EMC, PF_6_^−^-DMC-DEC, PF_6_^−^-DMC-2DEC, PF_6_^−^-MA-DEC, and PF_6_^−^-2MA-DEC, respectively. The white, red, cyan, gray, and purple balls represent H, O, F, C, and P atoms, respectively. The charge accumulation and depletion are colored in yellow and cyan [214]. Copyright 2023, Wiley–VCH. **b** Dynamic difference of the typical rocking-chair K-ion batteries (KIBs) and the designed potassium-based dual-ion batteries (K-DIBs). Dynamic advantage of the 0.5 M K electrolyte (right) in the designed K-DIBs [165]. Copyright 2023, Wiley–VCH. **c** Comparative analysis on the melting points and viscosity (25 °C) of typical carbonate solvents. LUMO and HOMO energy levels of the coordinated structures. Schematic illustration of interfacial chemistry on Li metal anode and graphite cathode via the energy-level-adaptive design of electrolytes [217]. Copyright 2024, Wiley–VCH

Based on the synergistic solvation strategy, researchers have developed a series of electrolyte solution systems by combining different solvents according to their characteristic moieties. Taking electrolyte of 1 mol/L LiPF_6_ dissolved in MA/DEC (8:2 by volume) (A8E2) as an example, the electrostatic potential mapping (ESP) for the solvents, PF_6_^−^ and PF_6_^−^-solvent complexes revealed that the negative moieties of the solvent molecules are concentrated near the O atoms and reach the extreme negative value on the O atom in the carbonyl group, while the positive charges are completely contributed by the H atoms of the alkyl groups (Fig. 10a) [214]. In contrast, the surfaces of PF_6_^−^ and PF_6_^−^-solvent complexes are completely negatively charged, promising that any parts of their surfaces have high Coulombic interactions with the positive electrode. The ESP results of PF_6_^−^ and solvent molecules imply that the bindings of PF_6_^−^ to the solvent molecule are mainly through the hydrogen bonding between the F atoms in PF_6_^−^ and the H atoms in alkyl groups. The batteries based on this electrolyte exhibited 93.8% of their room-temperature capacity at − 20 °C and can even work at − 70 °C.

The combination of cation-solvent co-intercalation chemistry and anion storage chemistry is a new design approach for designing DIBs that effectively circumvents the slow desolvation process [214, 215]. With a smaller Stokes radius and lower solvation energy, K^+^ endows electrolytes with enhanced tolerance toward low temperature with high ionic conductivity due to its reduced energy barriers (*E*_c_ and *E*_a_) on the anode/electrolyte interface, thereby promoting the cation/solvent co-intercalation at the anode side (Fig. 10b) [165]. Interestingly, the hydrogen titanate (HTO) anode exhibited excellent structural stability and cycling stability for this incredible K^+^/solvent co-intercalation mechanism despite the potentially violent volume changes involved during the continuous (de)intercalation process. Meanwhile, PTPAn cathode delivered fast surface-controlled anion-storage kinetics, ensuring excellent capacity retention at subzero temperatures. As a result, the HTO||PTPAn full cell intrinsically eliminated the rate limiting charge transfer process at subzero temperatures, maintaining high reversible capacity even when the temperature is reduced to − 60 °C. And the full cell showed improved cycling stability with a high capacity retention of 94.1% after 6000 cycles at − 40 °C.

As mentioned above, the electrode–electrolyte interphase has a significant impact on ionic conductivity, thus designing CEI/SEI with high anion/cation low-temperature transport kinetics via different strategies provides a new approach. For example, both FEMC and TFEP solvents show low melting points, relatively low viscosity, and high dielectric constant, which enable their possible application at low temperature [217]. Furthermore, as shown in Fig. 10c, the designed electrolyte by dissolving 1 mol/L LiFSI in FEMC/TFEP with a volume ratio of 7:3 v: v showed comparable HOMO levels with LiFSI but higher LUMO levels. On the one hand, this energy-level-adaptive electrolyte facilitates the formation of an organic-rich interphase containing N/P/S/F elements at the surface of the graphite cathode, which boosts anion transfer kinetics. On the other hand, the inorganic-dominant LiF-rich interphase at the anode side effectively restrained the growth of dendrites and retained the fast Li plating/stripping kinetics at low temperatures. The DIBs based on this electrolyte achieved a high capacity retention of 97.4% at − 40 °C compared to that at room temperature and showed no capacity decay after 300 cycles at 1 C under the operating temperature of − 20 °C.

### Strategies for Constructing More Reliable DIBs

**Fig. 11:**
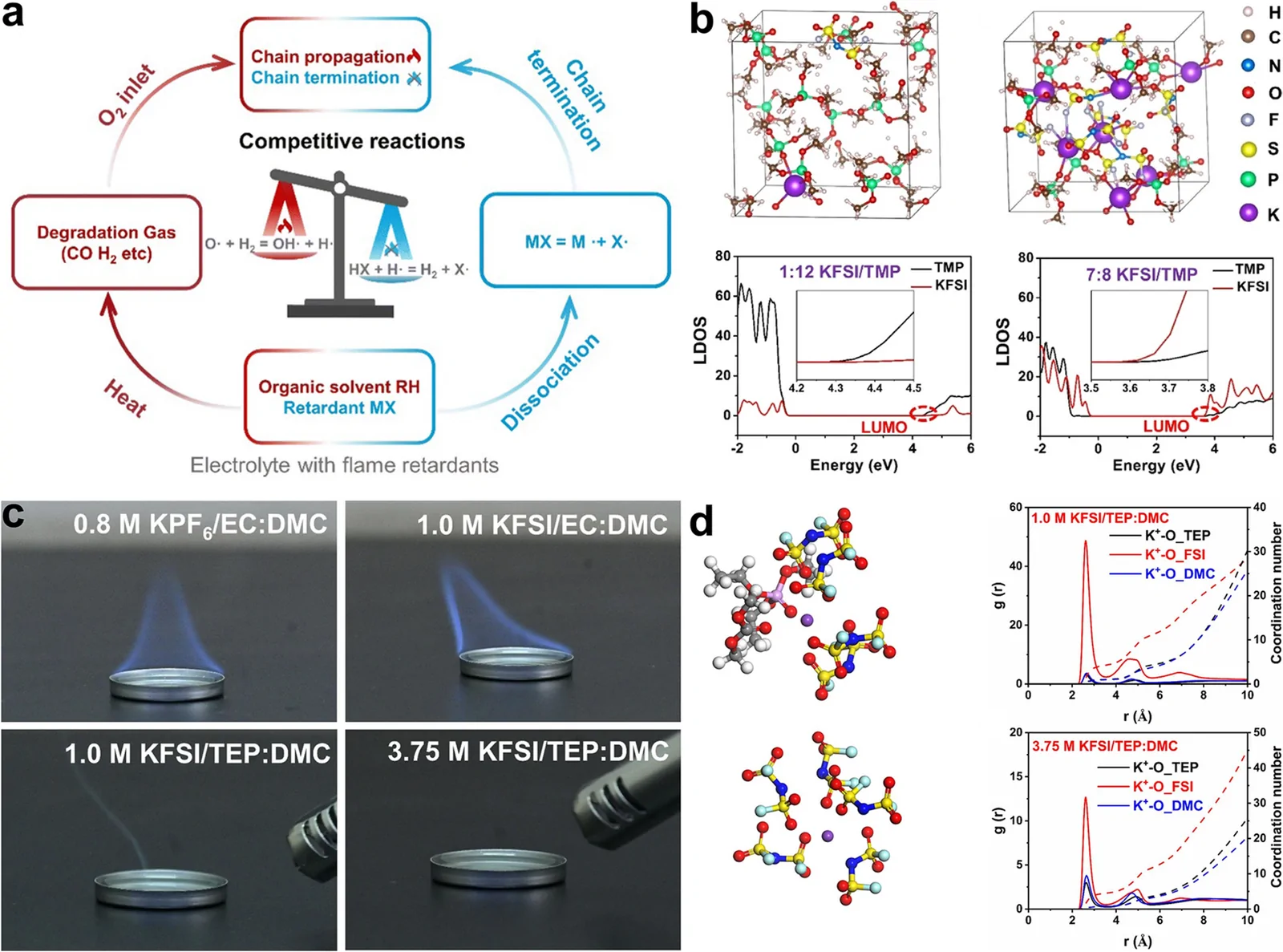
**a** Mechanisms of flame: the extinguishing or occurrence of gas flame depends on the competitive reaction process between radical chain propagation reactions (red) and chain termination reactions (blue) [225]. Copyright 2024, Royal Society of Chemistry. **b** Simulated electrolyte structure of KFSI/TMP with salt/solvent molar ratio of 1:12. LDOS of diluted KFSI/TMP electrolyte. Simulated electrolyte structure of KFSI/TMP with salt/solvent molar ratio of 7:8. LDOS of concentrated KFSI/TMP electrolyte. The insets are the magnified images at the edge of LUMO energy level [227]. Copyright 2020, American Chemical Society. **c** Flame test for the 0.8 M KPF_6_/EC: DMC, 1.0 M KFSI/EC: DMC, 1.0 M KFSI/TEP: DMC, and 3.75 M KFSI/TEP: DMC electrolytes. **d** K^+^ solvation structure, RDF and coordination number of the 1.0 M KFSI/TEP: DMC electrolyte. The K^+^ solvation structure, RDF and coordination number of the 3.75 M KFSI/TEP: DMC electrolyte [229]. Copyright 2025, Elsevier

The thermal hazards of rechargeable batteries are mainly caused by internal defects or external abuse, which are highly related to the flammable liquid electrolytes, raising serious safety concerns [95, 218, 219]. The development of nonflammable electrolytes is receiving widespread attention. Although solid-state electrolytes (SSEs) have been considered as competitive candidates for safe batteries due to their properties such as flame retardancy, oxidation resistance, and high energy density, these kinds of electrolytes still face challenges, including low ionic conductivity, high interfacial resistance, and high costs [220–222]. Moreover, unlike SSEs for traditional LIBs, which primarily focus on cation transport pathways, DIBs require additional attention to the design of transport pathways for anions with larger sizes, posing a huge challenge to the investigation of SSEs for DIBs [223, 224]. Therefore, it is urgent to develop new liquid flame-retardant electrolytes to enhance the safety and reliability of DIBs. Fire-extinguishing electrolytes utilizing the flame-retardant P, Cl, Br, and F-based solvents have been reported to enhance the battery safety [95]. Typically, as shown in Fig. 11a, trimethyl phosphate (TMP) and its derivative triethyl phosphate (TEP) are commonly used, which will release P scavenger to terminate the fire chain reactions [225].

TMP not only performs excellent flame retardancy but also delivers a lower HOMO value than that of EMC, indicating superior antioxidation ability of TMP compared to some commonly used carbonates [226]. The concentrated KFSI/TMP (molar ratios of 7:8) electrolyte showed a lower LUMO energy of KFSI than that of TMP (Fig. 11b), indicating more preferential reduction of KFSI at the Sn anode, which dominated the formation of SEI [227]. This designed electrolyte effectively improved the structural stability of SEI and inhibited the further decomposition of TMP solvent, boosting the cycling stability of DIBs, which showed good cycling stability with ∼81% capacity retention after 400 cycles. TEP has also displayed effectiveness in avoiding the occurrence of combustion with its excellent flame resistance [228]. As shown in Fig. 11c, both the dilute 1.0 mol L^−1^ KFSI in TEP: DMC (2:1 v/v) and the concentrated 3.75 M KFSI in TEP: DMC (2:1 v/v) electrolytes perform outstanding nonflammability even under sustained ignition [229]. In this electrolyte system along with the slat concentration increased from 1.0 to 3.75 M, the solvation structure of K^+^ undergoes a significantly shift: from a configuration with four FSI^−^, one TEP molecule, and one DMC molecule to dominated by five FSI^−^, indicating that increased concentration will enhance the coordination number of FSI^−^ in the solvation shell (Fig. 11d). Consequently, the DIBs assembled with the concentrated electrolyte showed an improved electrochemical stability with negligible capacity decay after 200 cycles at 200 mA g^−1^. As listed in Table 2, we summarized the current development of DIBs focusing on the cathode materials, electrolyte systems, working voltage, reversible capacity, rate capability, and cycling stability.

**Table 2:** A summary of recent development of DIBs concentrating on cathode material, electrolyte, voltage window, reversible capacity, rate capability, and cycling stability

Cathode	Electrolyte	Voltage (V)	Capacity (mAh g^−1^/mA g^−1^)	Rate (mAh g^−1^/mA g^−1^)	Cyclability (cycles/retention/mA g^−1^)	Refs
Graphite	1.7 M LiPF_6_ in FEC/EMC (4: 6 w/w) + 5 mM HFIP	4.0–5.2	80/ ~ 12	80/ ~ 12	50/ ~ 66/ ~ 12	[114]
Graphite	1 M LiPF_6_ in FEC/FEMC (3:7 vol%)	3.0–5.3	100/10	100/5000	5000/94.5/500	[115]
Graphite	4.0 M NaFSI in Cl-EMC	3.0–5.0	104.6/100	97.6/400	911/100/100	[116]
Graphite	5.2 M KFSI in TMS	3.0–5.4	83.4/100	34.8/400	300/100/100	[120]
Graphite	PP_14_NTF_2_	0.1–5.0	78.1/20	55.2/40	100/ ~ 75/40	[124]
Graphite	LiFSI-3TMS-3TTE (ratio in molar)	3.0–5.2	110/500	~ 110/200	1000/99/200	[136]
Graphite	LiFSI-2.5SUL-2.5TTE (ratio in molar)	3.0–5.2	~ 105/200	~ 95/2000	3000/95.7/1000	[49]
Graphite	3 M NaPF_6_ in EC/EMC(1:1 vol%) + FEC + TTE(50 vol%)	3.0–5.0	100/200	~ 80/3000	4000/96.4/1000	[137]
Graphite	3 M LiPF_6_ in EMC + 5%TMSP	3.0–5.0	101.3/100	98.2/2000	3000/92.5/1000	[138]
Graphite	1 M NaPF6 in EMC + 5 wt% FEC + 0.01–50 mg mL^−1^ NGO	3.0–5.1	95.5/100	82/5000	1500/96.2/1000	[147]
Graphite	1 M NaClO_4_ EC/DMC/EMC (1:1:1, v/v) + 5 wt% FEC	0.6–4.6	75/20	40/300	80/76/100	[153]
Graphite	1 M LiPF_6_ in EMC/sulfolane (SL)	3.0–5.4	100/100	91.6/800	2000/87.1/200	[148]
Graphite	3 M LiPF_6_ in EMC + 3% TTE	3.0–5.0	88.3/100	72/1500	1000/91.9/200	[155]
Graphite	3 M LiPF_6_ in EMC + 5 vol% FEC	3.0–5.1	~ 90/500	~ 84/3000	5000/85.1/500	[156]
Graphite	3 M LiPF_6_ in EMC + 0.5% LiDFOB	3.0–5.1	103/100	85.4/4000	6500/92.4/1000	[157]
Graphite	3 M LiFSI in FEC/FEMC (3:7 vol%)	3.0–5.1	95.8/200	88.9/2000	1000/92.3/200	[158]
Graphite	4 M LiPF_6_ in EMC	3.0–5.0	~ 80/100	~ 80/200	1000/83/200	[159]
Graphite	1 M LiPF_6_ in EMC	3.0–5.0	84.5/200	84.5/200	500/96/200	[160]
Graphite	4 M LiPF_6_ in EMC	3.0–5.0	81/200	81/200	1000/82.3/200	[161]
Graphite	4 M LiPF_6_ in EMC	3.0–5.0	~ 95/200	~ 95/200	8000/73.9/200	[162]
Graphite	1 M LiFSI in TFEP/FEMC (3:7 vol%)	3.0–5.2	101/500	91.2/1000	1000/100/1000	[166]
Nb_2_O_5_/graphene	2 M LiPF_6_ EC/DMC (1:1 vol%)	2.5–5.5	72/60	8/1200	250/100/200	[167]
WSSe/carbon skeleton	1 M NaClO_4_ in PC/EC (1:1 vol%)	1.0–4.2	123/500	65/4000	700/63/1000	[168]
CuPcNA-CMP	1 M LiPF_6_ in EC/DMC/EMC (1:1:1 vol%)	1.5–4.5	202.4/200	86.1/5000	110/50/200	[184]
Synthetic graphite	1 M LiPF_6_ in EMC	3.0–5.2	89.7/100	83/5000	300/ > 100/500	[174]
PAQDPZ	3 M LiFSI in TEGDME	1.5–4.3	224/100	129/5000	10,000/40.8/3000	[176]
Hybrid poly(CoL)n	1 M LiPF_6_ in EC/DEC (1:1 vol%)	1.6–4.5	192.13/50	104.1/5000	2000/89/5000	[175]
PQPZ	1 M NaPF_6_ in DIGLYME	1.0–4.0	270/138.5	146/2770	10,000/66.2/2770	[185]
P-PANI	1 M NaClO_4_ in EC/DEC (1:1 vol%) + 5vol% FEC	2.5–4.2	210/200	123/3000	200/68.1/200	[232]
DAQ-DPZ@C	3 M LiFSI in TEGDME	1.6–4.4	269/200	203/1000	1000/60.2/1000	[233]
pDHIC	2.35 M LiFSI in EC/DMC (1:1 vol%)	1.8–4.6	197/50	102/500	500/86.1/300	[186]
Graphite	5 M KFSI in EC/DMC (1:1 vol%)	3.2–5.25	98/50	47/500	300/89/100	[234]
VGNs	4 M LiPF_6_ in EMC + 2wt% VC	1.8–4.6	221/300	101/2000	2000/100/300	[171]
Ketjen black	1 M KPF_6_ in EC/PC (1:1 vol%)	1.5–4.6	232/50	110/2000	1000/100/300	[172]
N-LIDGs	1 M LiPF_6_ in EC/PC (1:1 vol%)	1.5–5.0	240/100	90/1000	200/90/100	[173]
Fc_2_NiNc	Li[FSA]-[C_2_C_1_im][FSA]	1.5–4.5	237/200	131.4/2000	1000/74.9/1000	[235]
Ti_3_C_2_T_x_ MXene	4 M LiPF_6_ in EMC + 1wt% VC, 2wt% DTD and 3wt% TMSP	1.0–5.0	310/200	92.9/1000	800/ > 100/200	[198]
PAD@MX	1.1 M NaPF_6_ in DEGDME	1.4–4.1	240/200	211/2000	800/92.6/1000	[115]
PAD@MX	2.2 M KPF_6_ in DEGDME	1.4–4.2	255/200	216/2000	400/71.8/1000	[115]
Graphite	4.0 M NaFSI in Cl-EMC	3.0–5.0	102/100	97.6/400	900/100/100	[119]
MSCG	4.0 M LiPF_6_ in EMC + 1wt% VC, 3wt% TMSP and 2wt% DTD	3.0–5.2	100.5/50	93/400	500/87.7/100	[88]
Graphite	[Na_0.56_K_0.44_][FSA]	2.5–4.7	108/20	~ 25/2000	-	[203]
PTPAn	1 M NaPF_6_ in G2	2.2–4.1	102.9/50	839/500	500/56.2/200	[210]
Graphite	1.2 M LiPF_6_ in DFEA + 10wt% FEC	3.0–5.2	~ 92/50	~ 77/8000	10,000/88/1000	[22]
Graphite	4.8 M LiFSI in FEA/DMC (8:2 vol%) + 1% LiPF_6_	3.0–5.1	90.8/500	102/3000	2000/93/500	[211]
Graphite	1 M LiPF_6_ in EFA/FEMC/FEC (6:3:1 vol%)	3.0–5.2	110/200	~ 70/1000	2000/100/200	[213]
Graphite	1 M LiPF_6_ in MA/DEC (8:2 vol%)	3.0–5.2	100/1000	~ 70/3300	1000/85.35/500	[214]
PTPAn	0.5 M KPF_6_ in DME	1.0–4.3	82/100	75.8/1000	20,000/86.7/500	[216]
PTPAn	0.5 M NaPF_6_ in DEGDME	1.0–3.9	100/100	45/10000	800/99.3/1000	[215]
PTPAn	0.5 M KPF_6_ in DME	1.0–4.3	90/50	~ 85/1000	6000/96.5/500	[165]
Graphite	1 M LiPF_6_ in FEMC/TFEP (7:3 vol%)	3.0–5.2	101/500	92.6/1000	1000/100/500	[217]
Graphite	6.6 M KFSI in TMP	3.0–5.2	96.2/200	86/500	400/81/300	[219]
Graphite	3.75 M KFSI in TEP/DMC (2:1 vol%)	3.0–5.35	99.2/100	51.7/500	200/100/200	[229]

### Strategies for Reducing Self-Discharge

Self-discharge represents a critical bottleneck hindering the practical deployment of DIBs, yet it has received disproportionately limited attention within standard laboratory coin-cell testing paradigms. Studies dedicated exclusively to elucidating this failure mode is scarce; instead, the mitigation of self-discharge is frequently reported merely as a collateral benefit in studies primarily targeting interfacial stabilization or capacity enhancement.

Since self-discharge is driven by the thermodynamic instability of high-stage GICs but enabled by surface parasitic reactions, constructing a stable passivation barrier is the most effective mitigation strategy. Recent studies highlight that a stable CEI functions as a dual-mechanism inhibitor. For instance, Kotronia et al. demonstrated that engineering the electrode interface via fluorinated binder chemistry can significantly reduce self-discharge rates [230]. This robust interface primarily suppresses the oxidative decomposition of the electrolyte to cut off the parasitic electron transfer pathway. Crucially, this chemical passivation translates into a kinetic barrier that effectively locks the metastable anions within the graphite lattice, counteracting the internal thermodynamic repulsion.

Simultaneously, for the anode, mitigating the spontaneous detachment of ions from the electrodes requires optimizing the electrolyte–electrode interaction. As demonstrated by Fan et al., replacing conventional organic solvents with ILs such as PP_14_TFSI offers a potent solution [231]. The inherent high viscosity and unique coordination environment of PP_14_TFSI creates a physical barrier that effectively blocks the self-deintercalation of active ions from the graphite anode. This viscous locking effect significantly retards the self-discharge process, enabling high coulombic efficiency (> 93%) and stable voltage retention even during extended rest periods.

Despite these promising electrode-level modifications, self-discharge remains a persistent challenge that demands prioritized attention within the DIB community. Ultimately, effectively resolving this issue at the device and pack levels will necessitate a holistic evolution in both material systems and manufacturing process capabilities to bridge the gap between laboratory research and practical application.

## Summary and Future Directions of DIBs

DIBs represent a new type of battery system that beyond traditional “rocking chair” batteries and have received great attention in the past few years. Based on its unique working mechanism, DIBs exhibit advantages such as high operating voltage and high energy density, high cathode diffusion kinetics, intrinsic safety of graphite cathode without oxygen release at high voltage, environmental friendliness and low cost brought by the absence of transition metals in the cathode [236–238]. However, as this review has discussed, there are still key challenges hindering their development, such as irreversible electrolyte decomposition at high operating voltage, solvent molecule co-intercalation, poor compatibility of electrolyte with both cathode and anode electrodes in a wide voltage window, mismatched kinetics between cathode and anode electrodes, low theoretical capacity of graphite cathode, poor low-temperature performance, and safety issues.

**Fig. 12:**
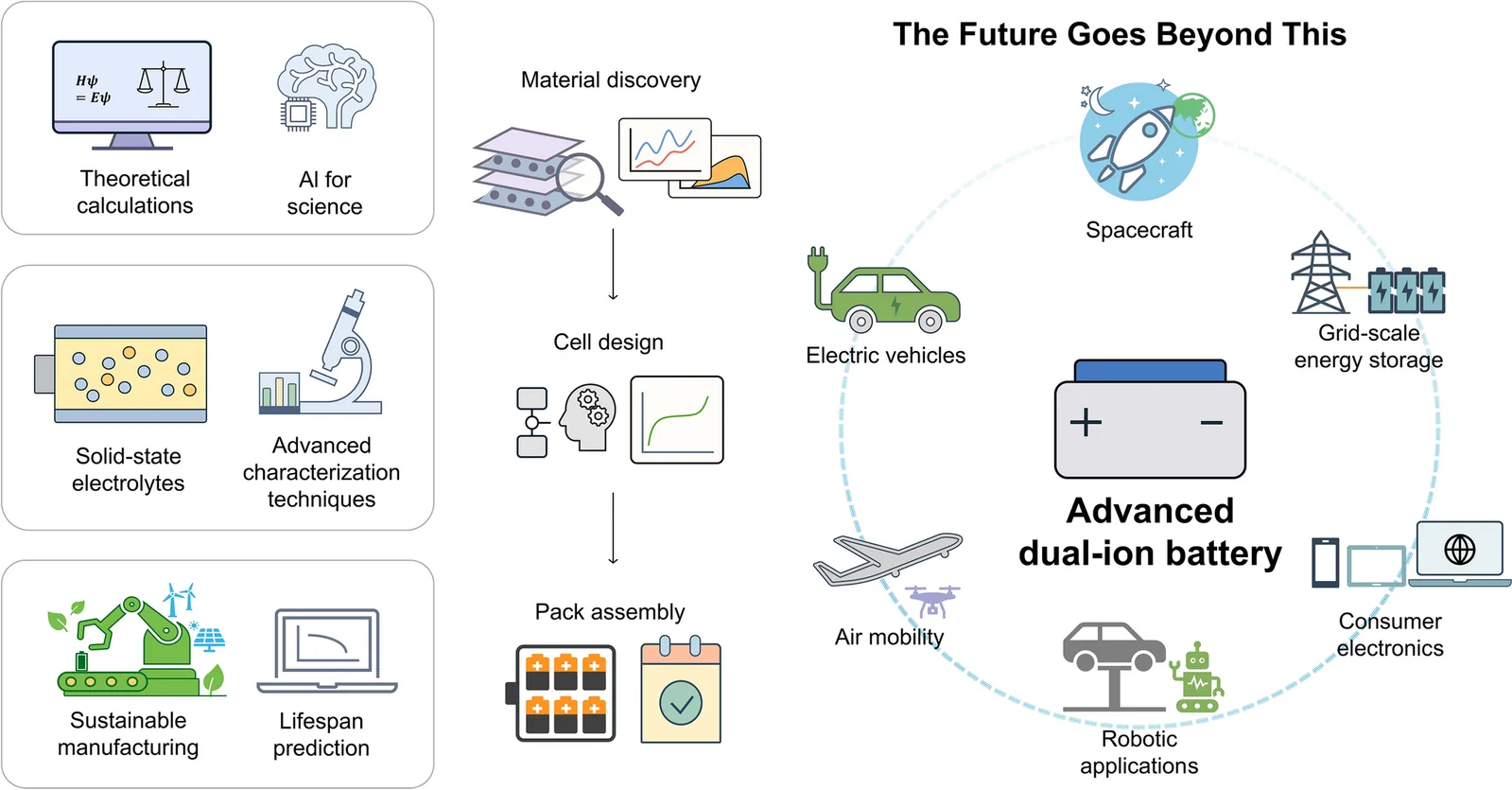
Advanced dual-ion batteries: enabling technologies and future applications

In recent years, researchers have achieved notable progress to address these issues, including designing high-voltage electrolyte systems, constructing stable electrode–electrolyte interphases, regulating anion solvation structures, tailoring anode materials with enhanced kinetics, developing high-capacity cathode materials, designing low-temperature resistant and flame-retardant electrolyte systems. In addition to the key scientific challenges and solutions that have been discussed in detail in this review, taking the rapidly increasing demands for energy storage technology into consideration, we highlight several key directions that need special attention for the future development of DIBs as follows (Fig. 12).

### Solid-State Electrolytes

SSEs provide the possibility of pursuing higher energy density and higher safety batteries due to their excellent oxidative resistance, thermal stability, and low flammability compared to organic liquid electrolyte [239]. Serve as a bridge for ion transport between cathode and anode, SSEs play a crucial role in solid-state batteries that directly determine the battery performance. SSEs are required to have high ionic conductivity and low electronic conductivity to ensure fast Li^+^ transport and reduce the self-discharge induced by electronic interference [240]. SSEs can be divided into two main categories based on their chemical composition: inorganic SSEs (oxides, sulfides, halides, nitrides, etc.) and polymer SSEs (PEO-, PVDF-, PEG-, PAN-based, etc.) [223–246]. The ionic conductivity of inorganic SSEs relies on Li^+^ migration within crystal lattice, while the Li^+^ conduction primarily depends on the movements of interchain, intrachain, and polymer chain. For DIBs, not only the transport of cations needs to be considered but also the transport pathways of larger anions require extra attention, which brings a significant challenge for the research of SSEs for DIBs.

It is necessary to design SSEs that can rapidly conduct both cations and anions simultaneously to ensure the optimal performance of DIBs. However, there are still a few all SSEs suitable for DIB that have been reported. Nevertheless, several gel polymer electrolytes (GPE), such as PVDF, PEO, PVA, PVDF‐HFP, etc., show high ionic conductivity and high ionic transference number in DIBs, indicating their practical applications in this field [224, 247–251]. Unfortunately, it is difficult to discover a polymer that can maintain stability both under the low reduction potential at the anode side and under the high oxidation potential at the cathode side. Typically, though PEO-based SSEs can passivate lithium metal, irreversible degradation at high voltage (> 4.0 V) makes it difficult for anion intercalation at the cathode. Thus, different strategies are required to design SSEs with excellent anion transport capability, oxidative resistance, and compatibility with both cathode and anode electrodes.

### Advanced Characterization Techniques

It is important to identify the evolution of materials and interfaces during electrochemical reactions, including phase evolution of both cathode and anode electrode materials, formation of electrode–electrolyte interphases, morphological and structural transitions, anionic solvation structures as well as charge compensation mechanisms. These processes usually occur during the extend cycles, which need the cooperation of different advanced characterization techniques.

There are many kinds of advanced characterization techniques that have been well applied in traditional secondary batteries, and can be referenced for DIBs [252]. 1) In situ X-ray diffraction (XRD), in situ Raman, neutron scattering techniques, and X-ray absorption spectroscopy (XAS), which can investigate the lattice structure and chemical conditions of the electrodes [253]. 2) Transmission electron microscope (TEM), cryo-TEM, and spherical aberration-corrected TEM, which provide visual information in atomic-scale and microstructural evolution of electrodes [254, 255]. 3) Focused ion beam (FIB), and X-ray computed tomography (CT), which can detect the internal microstructure of particles [256, 257]. 4) Nuclear magnetic resonance (NMR), time-of-flight secondary ion mass spectrometry (ToF–SIMS), and X-ray photoelectron spectroscopy (XPS), which can probe the chemical composition at the electrode–electrolyte interphases [258–260]. In the future, it is necessary to combine various characterization techniques to monitor the electrochemical reaction process of DIBs in real time, which facilitates exploring the basic electrochemical mechanisms represented by anion intercalation chemistry, promoting the understanding and leapfrog development of this emerging rechargeable battery system.

### Dual-Carbon Full Cells Design

In a dual-carbon full cell, negative/positive (N/P) ratio plays a crucial role in pursuing maximum material performance. A low N/P ratio (< 1) usually lead to precipitation of metallic Li/Na/K on the anode electrode due to the insufficient anode material, resulting in the formation of dendrites and safety issues. Conversely, as the N/P ratio increased over 1, indicating the excess of the anode material, which will lead to lower ICE and energy density [261–263]. Thus, it is important to balance the N/P ratio of the dual-carbon full cell by taking safety and energy density into consideration. Moreover, balancing the kinetics between the cathode and the anode is another key challenge, since the limited kinetics of the anode has significantly hampered achieving the high energy density and high power density. The structural design of anode materials for DIBs is expected to focus on the fast cation accommodation to ensure the compatibility with cathode at high current density. Therefore, the N/P ratio design of dual-carbon full cell also needs to consider the capacity retention of both the cathode and the anode under high rate conditions.

Beside the balance between cathode and anode, active cations and anions loss during the continuous charging/discharging is another key challenge for the dual-carbon full cells design. On the one hand, similar to the LIBs, DIBs will suffer from the Li loss at the anode side due to several factors such as the formations of unstable SEI derived by the decomposition of electrolyte, the accumulation of “dead Li”, irreversible adsorption in the defects of anode materials [264, 265]. These active Li losses not only induce a lower ICE and reduced actual energy density but also result in continuous depletion of Li reserves, leading to shorter service life and higher safety risks. On the other hand, the high operate voltage will induce more severe electrolyte oxidation at the cathode side, resulting in massive active anions loss. Meanwhile, irreversible solvent co-intercalation and adsorption by defects in graphite will also decrease the concentration of anions in electrolyte. Increasing the salt concentration is a promising approach to provide more active cations and anions as well as enhance the oxidation resistance of electrolyte and promote the formation of both CEI and SEI. Prelithiation is an effective strategy to improve the energy density and extend the service life of LIBs, which also feasible for DIBs [266–268]. Anode prelithiation can be achieved by electrochemical contact or chemical reduction reactions. As for the cathode, exploring the cathode preanionization additives and overanionizated cathode materials may be possible strategies to provide supplementary anion sources.

### Theoretical Calculations

Computational methods, particularly density functional theory (DFT) and molecular dynamics (MD) simulations, are emerging as indispensable tools for deciphering complex electrochemical phenomena in batteries at atomic and molecular scales [269, 270]. DFT provides quantum–mechanical insight into electronic structures, reaction energetics, and interfacial interactions. It allows researchers to evaluate bond strengths and energy barriers with high precision in processes such as ion desolvation. Complementarily, MD simulations capture the time evolution of atoms and molecules, making it possible to follow ion transport pathways, solvent motion, and the formation or disruption of solvation shells in real time. The integration of DFT and MD thus bridges electronic insight with dynamical behavior, yielding a more complete picture of electrochemistry in battery. This multi-scale perspective has become critical for unraveling the complex interplay among ions, solvents, and electrode surfaces, which is often challenging to probe experimentally.

In the context of DIBs, the judicious application of DFT and MD simulations can significantly accelerate the rational design of high-performance electrolytes by focusing on anion solvation structure modulation and interface electrochemical behavior. For instance, DFT can accurately predict the binding energies between various anions (like PF_6_⁻, TFSI⁻) and different solvent molecules, the strength of their solvation shells and the energy required for desolvation, which play a critical kinetic barrier for fast anion insertion at the cathode [271, 272]. Complementarily, MD simulations can track the dynamic behavior of these solvated anions, revealing diffusion pathways, quantifying ion mobility, and assessing the impact of solvent-ion interactions on bulk electrolyte conductivity [270, 273, 274]. By systematically screening various solvent combinations and electrolyte additives computationally, researchers can predictively identify formulations that lead to weaker anion solvation, thereby lowering desolvation barriers and promoting faster anion kinetics at the cathode, without compromising electrolyte stability. This integrated computational approach provides a powerful avenue for optimizing electrolyte formulations that balance high ionic conductivity with efficient anion desolvation, directly addressing kinetic limitations and enabling superior rate capabilities for future DIBs.

### AI for Science

The advent of AI and machine learning (ML) paradigms is fundamentally transforming scientific research, ushering in an era often termed “AI for science”. These powerful computational tools excel at processing vast datasets, uncovering hidden correlations, and predicting complex material properties with unprecedented speed and accuracy [275]. Importantly, AI for science goes beyond traditional forward design by enabling inverse design, in which the target material properties are specified first and the algorithms suggest suitable compositions or structures. Combined with high-throughput computational screening and data-driven experimental workflows, these approaches can greatly expand the efficiency of exploring chemical and structural spaces for materials discovery and optimization.

For DIBs, AI for science approaches hold promise for accelerating the design and discovery of both electrolytes and cathode materials. For electrolyte design, ML models can be trained on experimental and computational data to predict crucial properties like ionic conductivity, electrochemical stability window, viscosity, and desolvation energies of new solvent-salt-additive combinations [276–279]. This allows for high-throughput virtual screening of millions of potential electrolyte formulations, rapidly identifying promising molecular candidates with specific physicochemical properties tailored for diverse application scenarios. For instance, a knowledge-data dual-driven framework utilizing Transformer-based models and interpretable algorithms has been developed to predict melting points, boiling points, and flash points with high precision [280]. By screening over 130,000 molecules, this approach successfully identified promising non-flammable candidates, such as specific nitrile-based solvents, which balance wide temperature adaptability with high safety margins.

Simultaneously, AI can accelerate cathode material discovery for DIBs by predicting anion storage capacity, voltage profiles, rate capabilities, and structural stability based on elemental composition and crystal structure, while inverse design algorithms can suggest novel cathode architectures or compositions optimized for fast anion diffusion pathways and high energy density [281–283]. Furthermore, addressing the complexity of anion intercalation into graphitic channels requires capturing subtle interactions such as charge delocalization and specific ionic radii effects. Recent breakthroughs in machine learning potential, such as those employing Equivariant Graph Neural Networks (EGNNs) combined with explicit physical laws, now enable nanosecond-scale molecular dynamics simulations with ab initio accuracy. For instance, ReaxNet, an advanced machine learning potentials framework which combines EGNNs and polarizable charge equilibration model [284], can explicitly capture polarizable long-range interactions and reactive dynamics at interfaces, allowing researchers to simulate the distinct diffusion pathways and intercalation kinetics of novel anion structures. These AI-driven approach promises to dramatically reduce the time and cost associated with developing next-generation DIB components, paving the way for superior performance and commercial viability.

### Sustainable Manufacturing

Sustainable manufacturing has become a key requirement for the large-scale deployment of DIBs, linking material synthesis, cell processing, and end-of-life management to both carbon reduction and economic viability. At the materials level, recent studies highlight scalable and low-carbon routes for emerging DIB cathodes. For organic frameworks, aqueous polymerization, mechanochemistry, and bulk extrusion avoid toxic solvents and enable continuous synthesis [285, 286]. For nanostructured carbons and inorganic nanomaterials, template-free pyrolysis, spray-drying, and controlled graphitization provide tunable porosity with industrial compatibility [287–289]. In parallel, closed-loop recycling, which includes direct regeneration of graphite and porous carbons, binder systems designed for facile separation, and electrolyte-salt recovery, ensures resource efficiency and reduces lifecycle emissions [290].

At the system level, sustainable manufacturing emphasizes process and assembly innovations. Dry-electrode fabrication eliminates toxic solvents and high-energy drying, improving both environmental and economic metrics [291]. Cell-to-pack (CTP) integration reduces inactive components, enhancing material utilization at the pack level [292]. Meanwhile, the adoption of automation and robotic assembly lines, coupled with AI-driven process monitoring and adaptive control, enables high-throughput, consistent, and low-waste production. These combined advances form a coherent pathway toward the industrialization of DIBs under sustainability and circular economy principles.

### Lifespan Prediction

Ensuring the long-term safety and reliability of battery systems is another key for their widespread commercial adoption, particularly for emerging technologies like DIBs [293]. However, predicting battery lifespan presents significant challenges due to the multitude of complex, coupled, and often nonlinear degradation mechanisms that occur over thousands of charge–discharge cycles. These mechanisms, ranging from active material loss and electrolyte decomposition to SEI evolution and dendrite formation, are highly sensitive to operating conditions such as temperature, current rates, and depth of discharge. Traditional empirical aging tests are time-consuming and expensive, making it difficult to rapidly assess the durability and safety implications of new DIB chemistries and designs.

The future of DIB operation hinges on the integration of advanced lifespan prediction capabilities, largely driven by AI. By leveraging extensive cycling data, electrochemical impedance spectroscopy (EIS), voltage profiles, and temperature logs, ML models can be trained to identify subtle degradation fingerprints and accurately predict the remaining useful life (RUL) of a battery under various operating scenarios [294]. However, purely data-driven approaches may overlook the unique failure modes of DIBs arising from their distinct working mechanism, necessitating a deep coupling with physics-based degradation models to achieve precise lifespan prediction. Recent advances have established specialized frameworks for this purpose, exemplified by the modified Doyle-Fuller-Newman (DFN) model developed by Innocenti et al., which specifically captures the macroscopic electrochemical dynamics of DIBs by simulating the critical variations in bulk electrolyte salt concentration and internal gradients during charge–discharge cycles [295]. This macroscopic perspective is complemented by the finite strain framework proposed by Roque et al., capable of explicitly quantifying structural degradation. By employing a phase-field fracture approach, this model elucidates how the massive volume expansion (> 100%) and diffusion-induced stress fields from bulky anion intercalation drive crack nucleation and propagation in graphite cathodes [296]. By integrating these physics-derived electrochemical and structural models into AI algorithms, proactive battery management systems (BMS) can impose dynamic charging strategies that optimize lifespan and prevent premature failure [297]. Moreover, real-time health monitoring and precise lifespan prediction are crucial for enhancing safety by forecasting potential degradation-induced risks, ensuring the long-term reliability and economic viability of DIBs in diverse applications.

In summary, DIBs represent an exciting frontier in the pursuit of safe, high-voltage, and sustainable electrochemical energy storage. By leveraging anion intercalation chemistry in graphite and other layered hosts, DIBs integrate the benefits of high operating potential, structural stability, and low-cost electrode materials. Substantial progress has been achieved in electrolyte molecular engineering, interfacial stabilization, and electrode material development, which has enabled improvements in voltage window, cycling stability, and rate capability. Nevertheless, several fundamental challenges remain unresolved. For example, electrolyte decomposition at high voltage, solvent co-intercalation, and instability of electrode–electrolyte interphases continue to limit long-term stability. Kinetic asymmetry between cathode and anode processes, restricted anion storage capacity of graphite, and reduced performance under low-temperature or high-rate conditions further constrain practical energy density. Safety concerns associated with gas evolution and thermal runaway also require careful consideration. Recent studies have demonstrated that electrolyte design, through molecular functionalization, localized high-concentration systems, and weakly solvating environments, plays a central role in enabling reversible high-voltage operation. Interfacial stabilization strategies have facilitated the formation of protective CEI, mitigating continuous side reactions at elevated potentials. Advances in electrode materials, including graphite derivatives, polymer-based frameworks, and hybrid carbon architectures, have broadened the scope for improving anion storage capability and cycling stability. Looking forward, the path to practical DIBs requires progress in several directions. In situ and operando characterization techniques are indispensable for revealing the dynamic processes of anion solvation, interfacial evolution, and structural transformations. Multiscale theoretical simulations are needed to provide molecular-level insights into intercalation energetics and electrolyte stability. Device-level engineering, such as optimization of electrode architectures and dual-carbon full-cell configurations, will be crucial for bridging laboratory progress with practical deployment.
